# Systematic review on the laboratory methodology for conducting wastewater and environmental surveillance for *Salmonella*

**DOI:** 10.3389/fpubh.2026.1755256

**Published:** 2026-02-23

**Authors:** Lucky Sangal, Vishesh Sood, Karin Haar, Takana Mary Silubonde, Yuka Jinnai, Suman Rijal

**Affiliations:** 1 Immunization & Vaccine Development Unit, World Health Organization South-East Asia (WHO SEARO), New Delhi, India; 2 Department of Communicable Disease, World Health Organization South-East Asia (WHO SEARO), New Delhi, India; 3 Research and Innovation Department, World Health Organization South-East Asia (WHO SEARO), New Delhi, India; 4 Health Emergency Information and Risk Assessment, Health Emergencies Programme, World Health Organization South-East Asia (WHO SEARO), New Delhi, India

**Keywords:** laboratory methodology, low- and middle-income countries (LMICs), *Salmonella*, *Salmonella Typhi*, wastewater and environmental surveillance (WES)

## Abstract

**Introduction:**

Wastewater and environmental surveillance (WES) is a valuable supplementary tool to clinical surveillance for infectious diseases, especially in low- and middle-income countries. This systematic review evaluates laboratory methods for detecting *Salmonella* spp. in wastewater and contaminated surface waters, focusing on methodological diversity, feasibility, and the need for standardized protocols.

**Methods:**

The review was performed using protocol registered with PROSPERO (ID: CRD42024573052) following PRISMA 2020 guidelines. The review was funded by the European Commission’s Health Emergency Preparedness and Response Authority (HERA) and the World Health Organization (WHO). Search was conducted in PubMed, EMBASE, and Web of Science (last update: May 31, 2025). Studies describing sampling and laboratory methods for *Salmonella* detection in wastewater or contaminated surface waters were included. Exclusion criteria were incomplete methodology, non-peer-reviewed status, or non-English publication. Data extraction and quality assessment were performed independently by two authors. Results were synthesized narratively due to high methodological variability.

**Results:**

Of 2,007 records, 94 studies from 36 countries met inclusion criteria. Grab sampling was most common, followed by trap and composite sampling. Detection methods included culture, PCR, and sequencing. Six methodological pathways were identified. Fewer than 14% of studies reported comprehensive quality control. Substantial heterogeneity in sampling, handling, and testing protocols affected reproducibility and comparability.

**Discussion:**

Evidence generated was constrained due to inconsistent reporting of quality control and validation criteria. Most studies lacked critical methodological details for reproducibility and scale-up. Harmonized, context-adapted protocols and minimum reporting standards are needed, along with leveraging existing surveillance infrastructure for rapid implementation of *Salmonella* WES in diverse settings.

**Systematic review registration:**

https://www.crd.york.ac.uk/PROSPERO/view/CRD42024573052, identifier PROSPERO (CRD42024573052).

## 1 Introduction

Wastewater and environmental surveillance (WES) is well-established for poliovirus surveillance as a core part of the Global Polio Eradication Initiative (1–4), and gained further prominence during the COVID-19 pandemic as a valuable tool for understanding the disease burden in communities (5) The success of WES in COVID-19 has sparked interest in its use to monitor other pathogens of public health concern (6). Prioritization of pathogens for WES remains a fundamental question for optimizing resource utilization and ensuring operational flexibility and adaptability in the event of outbreaks caused by new pathogens (7–9). Proposed prioritization frameworks emphasize several key factors for successful WES adaptation, including the public health significance of the pathogen, the usefulness of WES data for public health actions, and the analytical feasibility of conducting WES (8, 9). Prioritizing a pathogen within this framework helps align WES efforts with broader public health goals.

*Salmonella* infections, particularly with typhoid and paratyphoid serovars, continue to pose a substantial burden on global public health systems, and remain a significant challenge in low- and middle-income countries (LMICs), including within the World Health Organization’s South-East Asia Region (SEAR) (10, 11). Non-typhoid and non-paratyphoid *Salmonella* serovars. Infect both humans and various animals, making them a significant concern for zoonotic transmission and for both animal husbandry and the food industry (12, 13). Asymptomatic carriers and subclinical infections play a key role in maintaining the transmission chain of *Salmonella* infections (14, 15). Therefore, accurately determining the true prevalence of *Salmonella*-related diseases requires supplementing clinical surveillance with serosurveys or contact tracing during outbreaks (16, 17). However, current methods for additional surveillance have limited sensitivity. For instance, the clinical diagnosis of typhoid and paratyphoid often relies on non-specific Widal tests or blood cultures, both of which have low sensitivity due to suboptimal sampling times post-incubation period (18, 19). Despite the World Health Organization not recommending the Widal test, it remains widely used in clinical practice in our region. In addition to its limited sensitivity, the Widal test is prone to cross-reactivity with other pathogens, further reducing its diagnostic specificity. Additionally, estimating HlyE IgG antibodies using ELISA is the preferred method for serosurveys on typhoid and paratyphoid prevalence; however, HlyE antibodies can exhibit cross-reactivity, as many other bacteria also express HlyE (20, 21). As a result, there is a need to establish supplementary tools to accurately estimate the true prevalence of the infections caused by *Salmonella* Typhi and Paratyphi.

Since *Salmonella* is present in wastewater due to shedding in the feces of both symptomatic and asymptomatic individuals, depending on the stage of infection, WES has proven effective in evaluating its community burden in endemic countries, complementing existing clinical surveillance and serosurveys (22–24). Besides assessing community-level disease burdens, WES can also generate data on circulating strains and antimicrobial resistance—provided the bacteria can be cultured—which directly informs vaccination and antimicrobial resistance (AMR) strategies, offering significant public health benefits for *Salmonella* monitoring (25, 26). Therefore, the public health importance of *Salmonella* and the utility of WES make it a priority pathogen for WES implementation (27).

The primary challenge of *Salmonella* WES lies in analytical feasibility, due to the heterogeneous and variable factors outside the laboratory that affect sample collection and quality. Unlike high-income countries with centralized and closed sewage systems, most *Salmonella* Typhi and Paratyphi-endemic countries, particularly in the SEAR region (24, 28, 29), frequently rely on decentralized, informal, or mixed drainage networks, including open drains, septic tanks, and combined sewer-stormwater systems, which are often poorly maintained and vulnerable to contamination during monsoons (30, 31). Thus, the pre-examination factors such as variability in infrastructure, flow dynamics, and ambient conditions complicate sample collection and pathogen recovery and demand context-specific adaptations to sampling and testing protocols. Laboratory capacity constraints, including cold chain logistics and molecular diagnostics standardization and result interpretations, further limit the applicability of WES protocols (28, 29, 32).

Inherent variability in sampling site characteristics, public health goals, and laboratory capacity underscores the urgent need to develop harmonized methodologies that are scientifically sound, operationally practical, and adaptable to diverse infrastructure contexts, particularly in low- and middle-income countries. This systematic review was conducted to evaluate the scientific and operational feasibility of laboratory methods for detecting *Salmonella* in wastewater, aiming to guide the development of harmonized, context-specific field and laboratory protocols that can support regional public health goals like integrated disease surveillance and inform the deployment of typhoid conjugate vaccine (TCV) in endemic areas and LMICs. The review assessed the completeness of methodological reporting; such as site selection, sample handling, and quality control to identify critical gaps in methodological reporting that might impede reproducibility and scalability. The identified methodologies were further categorized as pathways to match protocol steps with the wastewater sampling and socio-economic status of reporting countries. Finally, the review also identified the primary public health domains that researchers utilize to develop a framework for aligning surveillance objectives with laboratory capacities and infrastructure realities.

## 2 Methods

### 2.1 Study design

This qualitative systematic review was designed and reported in accordance with the Preferred Reporting Items for Systematic Reviews and Meta-Analyses (PRISMA) 2020 guidelines (33). A detailed protocol outlining the objectives, eligibility criteria, and methodological approach was developed before the initiation of the review and registered with the International Prospective Register of Systematic Reviews (PROSPERO) on August 5, 2024 (registration ID: CRD42024573052). The PICOS framework was adapted to develop a research question and inclusion and exclusion criteria were defined to align with PICOS framework (Supplementary Table 7). Based on the PICOS framework, the objective of this systematic review was to map the diversity in the laboratory workflows and thematic domains used for the detection of *Salmonella* spp. from wastewater and wastewater-impacted surface waters, and to synthesize these findings into evidence-based heuristic framework for resource planning. Further, the systematic review also aimed at evaluating the completeness of the reporting of laboratory methodologies and work on the recommendations to ensure reporting of reproducible methodology.

### 2.2 Search strategy

A comprehensive literature search was conducted to identify peer-reviewed studies describing laboratory methodologies for the detection or isolation of *Salmonella* spp. From wastewater and wastewater contaminated surface waters. The scope of methodologies was kept at species levels as isolation and characterization at species level can be extended to specific sub-species. The initial search was conducted on September 10, 2024, using three major scientific databases: PubMed, EMBASE, and Web of Science. It was updated on May 31, 2025, to include recent publications. The specific combination of databases was chosen because it achieves a 90–95% recall rate in over 80% of reported systematic reviews (34). Google Scholar was not included in the search strategy to improve the recall rate further as Google search uses proprietary algorithms that can result in variation in search results based on time, place, and person, thereby making Google Scholar an unreliable tool when repeatability is required (35, 36). The search strategy was designed to retrieve studies relevant to *Salmonella* Typhi surveillance in environmental matrices, with a focus on wastewater and surface waters. Briefly, a naïve search was conducted on PubMed after identifying relevant MESH and MAJR terms for WES of *Salmonella* spp. The query used was - “*Salmonella*”[Mesh] AND (“Wastewater-Based Epidemiological Monitoring” [Mesh] OR “Environmental Monitoring”[Mesh] OR “Sewage/microbiology”[MAJR] OR “Wastewater/microbiology”[MAJR]). The easyPubMed package in R was used to import the naïve search results, which were analyzed with the litsearchr package in R to identify keywords in an unbiased manner (Supplementary Figure 1; Supplementary Table 3) (37, 38). The identified keywords were used to create PubMed search queries using Boolean operators and wildcard symbols (e.g., *). The search query was optimized to evaluate its sensitivity against a benchmark set of 16 studies on *Salmonella* spp. (Supplementary Table 4). The search terms were refined until all 16 benchmark studies were captured (Supplementary Figure 2; Supplementary Table 5). Once 100% sensitivity was achieved for the benchmark studies, the final PubMed query was translated into EMBASE and Web of Science formats using the polyglot application (39). The final search queries for each database are provided in (Supplementary Table 6).

### 2.3 Systematic review process

The final search was conducted in May 2025, and results were imported into the Covidence platform, which automatically removed duplicates. Remaining duplicates were manually reviewed and excluded. Title and abstract screening was then performed, with inclusion and exclusion criteria detailed in Supplementary Table 7. The proportional agreement for the screening step by the authors is provided in Supplementary Table 13. Two authors independently reviewed the studies, resolving conflicts by consensus among all authors. Full texts were retrieved for eligible studies and screened again against the criteria. The Covidence platform was also used to prepare templates for data extraction and completness of reported methodolgy assessment, as described in Supplementary Tables 8, 9. Data extraction was performed independently by two authors, with disagreements resolved by consensus. Supplementary Tables 1, 2 present the 2020 PRISMA checklist and PRISMA Abstract checklist for this systematic review, respectively.

### 2.4 Data analysis and visualization

A narrative synthesis was conducted due to the variability in study designs, sampling strategies, and laboratory methods. Data visualization involved subgroup analysis based on the methodological areas (e.g., sampling, processing, testing), and patterns were identified across the studies Further, an exploratory analysis was performed to understand commonalities between reported methods to identify major pathways and to identify thematic domains and their interconnectedness in the study dataset of selected manuscripts.

Python (version 3.12.7) was used within the Spyder IDE (version 6.0.7) to perform data analysis and visualization, using a collection of specialized libraries. Pandas handled data import and transformation; NumPy supported numerical calculations; and SciPy was used for estimating Jaccard distances and performing chi-squared tests. The silhouette score was calculated with Scikit-learn, while NetworkX enabled network analysis. GeoPandas managed geospatial polygon data for countries, Matplotlib produced standard plots, Seaborn generated heatmaps, and UpSetPlot was used to create UpSet diagrams.

Geographical mapping of studies was conducted using cultural raster map shapefiles obtained from Natural Earth and analyzed with the GeoPandas library in Python. Since some studies reported using multiple methods or samples, the methods were identified to aid analysis. To find commonalities among the methods, pathway analysis was performed. During this process, methods were clustered using the Jaccard distance approach based on factors such as the economic status of countries, sample types, and the presence or absence of specific protocol steps. For simplicity, the LMIC classification included all countries from the LIC (low-income countries), LMIC, and UMIC (upper middle-income countries) categories. The samples analyzed included grab samples, trap samples, and composite samples. Protocol steps included processing, culture, biotyping (using biochemicals and other biotyping techniques), serotyping (via the Kauffman-White scheme and PCR), antimicrobial susceptibility testing, genotyping (using molecular assays and ARGs), and genomics methods, including targeted sequencing, whole-genome sequencing, and metagenomics. For understanding the commonalities in the reported methodologies, the identified methods were clustered. For clustering, qualitative descriptors were converted into a binary matrix. Each unique protocol step was defined as a binary variable with ‘explicit encoding’ of the presence and absence of the step as ‘1’ and ‘0’, respectively. To empirically identify common methodological pathways, a hierarchical clustering on the binary protocol matrix was performed. Jaccard distance was used to measure the dissimilarity between the studies as it prioritizes the presence of shared features while ignoring the shared absence. Average linkage was used to minimize the variance between the clusters. The optimal number of clusters was determined through the silhouette score and the elbow method. The results were reported as the probability of presence of a step in the pathway. To verify that the pathways identified were not a result of poor reporting, a stability analysis was performed with respect to the completeness of methodology reporting (Supplementary Table 10; Supplementary material 2). First, the reporting scores of pathways excluded from clustering was compared with those of clustered methods using a Welch’s t-test to justify their exclusions from the pathway clustering. The stability of clustering of retained methods was tested using the one-way ANOVA to test variability in reporting scores across pathways. The one-way ANNOVA was also repeated at the study level by aggregating the scores by Paper ID. Finally, the internal cluster stability was quantified using a bootstrap approach (*n* = 1,000 iterations) to calculate the coefficient of variation (CV) of quality scores. To further explore the thematic domains in the selected papers, two authors identified the domains based on the titles, keywords, and abstracts of the selected studies and any disagreement was resolved based on all authors conensus. Eight thematic domains were recognized: (A) outbreak detection/investigation, (B) disease prevalence, (C) AMR prevalence, (D) mechanisms of AMR, (E) wastewater monitoring, (F) environmental health, (G) One Health, and (H) method validation. Some studies encompassed more than one domain. The co-occurrence of domains was calculated using NumPy. To visualize the core domains and their connections to other domains, the co-occurrence matrix was interpreted as an undirected weighted graph with NetworkX. The network comprises nodes representing individual domains, with node size proportional to the number of studies in each domain. Edges indicate co-occurrences between domains, with edge width reflecting the strength of these co-occurrences. Additionally, a force-directed spring layout was used to position strongly related domains based on the study dataset closer together.

### 2.5 Reporting bias

The review aimed to eliminate bias during both the searching and review stages. To minimize search bias, multiple databases were utilized, and the search string was refined through unbiased keyword selection and by evaluating the search strategy against a set of benchmark studies. For study selection bias, two authors independently reviewed the abstracts during the screening process to determine eligibility for full-text review, and two authors independently conducted the data extraction.

## 3 Results

### 3.1 Literature search results

**Figure 1:**
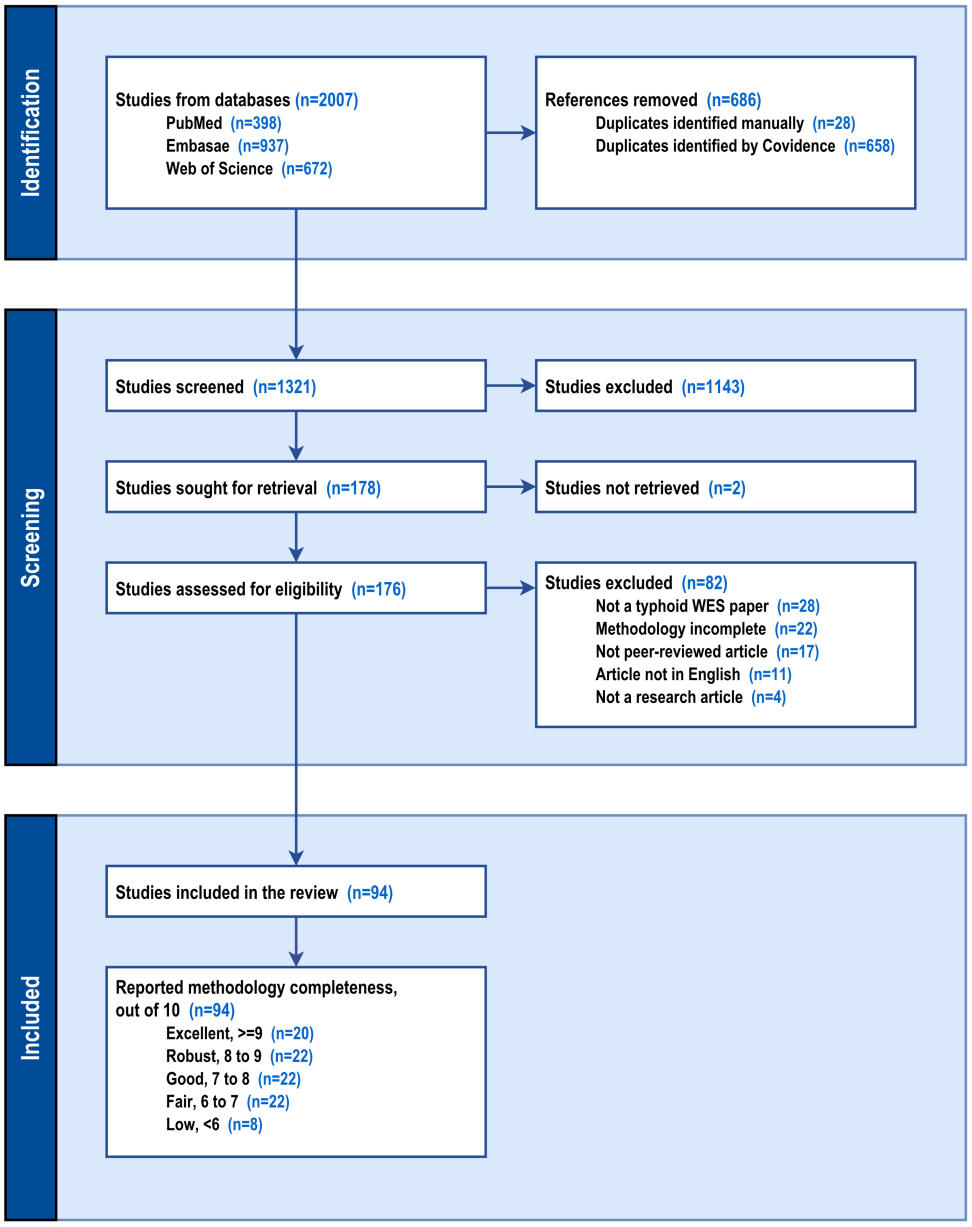
PRISMA flow diagram for database searches, deduplication, screening, retrieval, exclusion, inclusion, and quality-based classification of studies on WES for *Salmonella* spp.

A total of 2,007 articles were identified across PubMed, Embase, and Web of Science. After removing 686 duplicates, 1,321 records were screened by title and abstract, resulting in 1,143 exclusions. Of the 178 full-text articles assessed, 94 met the eligibility criteria and were included in the review (22–24, 26, 28, 29, 32, 40–126). Studies were excluded for reasons such as incomplete methodology, non-peer-reviewed status, or publication in a language other than English. Included studies were further classified by methodological quality assessment into five categories: excellent (*n* = 20), robust (*n* = 22), good (*n* = 22), fair (*n* = 22), and low (*n* = 8). The PRISMA workflow for the systematic review is presented in Figure 1. The extracted data and quality assessment data are provided as Supplementary materials 1, 2, respectively. As some studies used multiple samples and multiple methods, the extracted data were further refined to obtain the methods used for each sample in different studies. This resulted in the identification of 102 methods. Table 1 summarizes the sample types and methodology used by the included studies.

**Table 1:** Methodologies (*n* = 102) extracted from included studies (*n* = 94) for WES for *Salmonella* spp.

Paper ID (Reference)	Sample	Processing	Culture	Enumeration	Biotyping	Serotyping	AST	Pheno. other	Bacteriophage	Molecular	Mol. ARG	Mol. other	Sequencing
Paper_001 (24)	G	✓								✓			
	T		✓							✓			
Paper_002 (40)	T	✓	✓	✓						✓			
Paper_003 (41)	G	✓	✓		✓		✓				✓		
Paper_004 (42)	G	✓											✓
Paper_005 (43)	G	✓	✓		✓	✓	✓						
Paper_006 (44)	T		✓							✓			✓
Paper_007 (45)	T		✓		✓	✓	✓						
Paper_008 (46)	C	✓	✓		✓	✓	✓						
Paper_009 (47)	G	✓	✓		✓		✓						
Paper_010 (48)	G	✓	✓		✓	✓							
Paper_011 (49)	T		✓							✓			✓
Paper_012 (50)	G	✓											✓
Paper_013 (51)	G	✓	✓		✓					✓			
Paper_014 (52)	G	✓	✓		✓	✓	✓				✓	✓	✓
Paper_015 (53)	G	✓	✓		✓	✓	✓				✓		
Paper_016 (54)	G	✓	✓		✓		✓				✓		
Paper_017 (55)	G	✓	✓		✓	✓	✓						
Paper_018 (56)	G	✓	✓				✓				✓		✓
Paper_019 (57)	G		✓			✓	✓			✓		✓	
Paper_020 (58)	G	✓	✓		✓	✓	✓						
Paper_021 (59)		✓	✓		✓	✓	✓				✓		✓
Paper_022 (60)	G		✓		✓	✓	✓					✓	
Paper_023 (61)	C	✓								✓			
Paper_024 (62)	C	✓	✓							✓			✓
Paper_025 (63)	C	✓											✓
Paper_026 (64)	G	✓					✓			✓	✓		✓
Paper_027 (65)	G		✓		✓		✓				✓		
Paper_028 (66)	G	✓	✓			✓	✓			✓		✓	
Paper_029 (67)	G		✓				✓	✓		✓	✓		✓
Paper_030 (68)	G	✓							✓				
Paper_031 (69)	G	✓											✓
Paper_032 (70)	T	✓											✓
Paper_033 (28)	G	✓								✓			
Paper_034 (71)	G	✓	✓		✓		✓						
Paper_035 (73)	G	✓	✓		✓	✓				✓			
Paper_036 (72)	G	✓	✓		✓	✓	✓	✓		✓		✓	
Paper_037 (74)		✓								✓			✓
Paper_038 (75)	G	✓	✓		✓					✓			
Paper_039 (76)	G		✓		✓		✓						✓
Paper_040 (77)	G	✓								✓			
Paper_041 (78)	G	✓											✓
Paper_042 (79)	G		✓										
Paper_043 (80)	G	✓	✓		✓								
Paper_044 (81)	S	✓	✓		✓		✓			✓		✓	
Paper_045 (82)	C	✓								✓			
Paper_046 (83)	G	✓	✓							✓			
Paper_047 (84)	G		✓				✓						✓
Paper_048 (85)	T		✓							✓			
	G	✓								✓			
Paper_049 (87)	G	✓	✓		✓		✓					✓	
Paper_050 (88)	G	✓											✓
Paper_051 (89)	T		✓			✓	✓	✓			✓	✓	
Paper_052 (90)	G	✓	✓		✓					✓			✓
Paper_053 (91)	G	✓	✓		✓		✓				✓		✓
Paper_054 (86)	C	✓	✓							✓			✓
Paper_055 (92)	G	✓	✓		✓		✓						
Paper_056 (93)	G		✓		✓		✓						
Paper_057 (94)	G	✓	✓		✓		✓			✓			
Paper_058 (95)	C		✓		✓		✓			✓			✓
Paper_059 (29)	G	✓								✓			
	T									✓			
Paper_060 (96)	G	✓	✓		✓		✓						
Paper_061 (97)	G	✓	✓		✓		✓						
Paper_062 (98)	G	✓	✓			✓		✓		✓			✓
Paper_063 (32)	G	✓								✓			
	T		✓							✓			
Paper_064 (99)			✓		✓	✓	✓				✓		
Paper_065 (100)	G		✓		✓		✓			✓	✓		✓
Paper_066 (101)	G	✓	✓		✓		✓			✓			
	T		✓		✓		✓			✓			
Paper_067 (102)	G	✓											✓
Paper_068 (103)	G	✓								✓		✓	
	G	✓	✓		✓	✓	✓						
Paper_069 (104)	C	✓								✓			
Paper_070 (105)	T	✓	✓		✓	✓	✓						
Paper_071 (106)	T	✓	✓		✓	✓		✓					
Paper_072 (107)	T		✓		✓	✓		✓					
Paper_073 (108)	G		✓				✓				✓		
Paper_074 (110)	G	✓								✓			
Paper_075 (109)	G	✓							✓				✓
Paper_076 (111)	G	✓								✓			
Paper_077 (112)	G	✓								✓	✓		
Paper_078 (113)	G	✓	✓	✓	✓		✓						
Paper_079 (114)	G	✓	✓		✓	✓	✓				✓	✓	
Paper_080 (115)	G	✓								✓			
Paper_081 (116)	G	✓	✓	✓		✓	✓			✓		✓	
Paper_082 (117)	G	✓								✓			
Paper_083 (118)	G		✓		✓	✓	✓			✓			
Paper_084 (119)			✓		✓								✓
Paper_85 (120)	G	✓								✓			
	T	✓	✓							✓			
Paper_86 (22)	G	✓								✓			
	T	✓	✓							✓			
Paper_87 (121)	T		✓										
Paper_88 (122)	G		✓		✓		✓						
Paper_89 (123)	G		✓		✓		✓						
Paper_90 (124)	G		✓		✓	✓				✓		✓	✓
Paper_91 (125)		✓								✓			
Paper_92 (26)	C	✓	✓	✓	✓					✓		✓	
Paper_093 (23)	G	✓	✓		✓	✓	✓			✓		✓	
Paper_094 (126)	G	✓	✓				✓			✓	✓		

The complete extracted dataset is provided as Supplementary material.

Sample type: G, Grab sample; T, Trap sample; C, Composite sample; S, Sewage sludge; blank, no sample specified. Methodology: ✓, step used in the reported method; blank, step not used in the reported method.

Sample, Sample type; Processing, Sample processing steps performed before testing; Culture, Bacterial culture step; Enumeration, Bacterial enumeration (quantification); Biotyping, Biochemical tests used for bacterial characterization; Serotyping, Serotyping performed using serum (serological) agglutination assays and/or PCR-based serotyping; AST, Antimicrobial susceptibility testing; Pheno. Other, Other phenotyping methods used; Bacteriophage, Bacteriophage-based protocols/methods; Molecular, Molecular detection methods used for bacterial targets (e.g., PCR, RT-PCR, and related variants); Mol. ARG, Molecular detection of antimicrobial resistance genes (ARGs) (e.g., PCR, RT-PCR, and related variants); Mol. Other, Other molecular methods (e.g., pulsed-field gel electrophoresis (PFGE)); Sequencing, Sequencing-based methods, including targeted sequencing, whole-genome sequencing (WGS), or metagenomics.

### 3.2 Geographical distribution of reported studies

**Figure 2:**
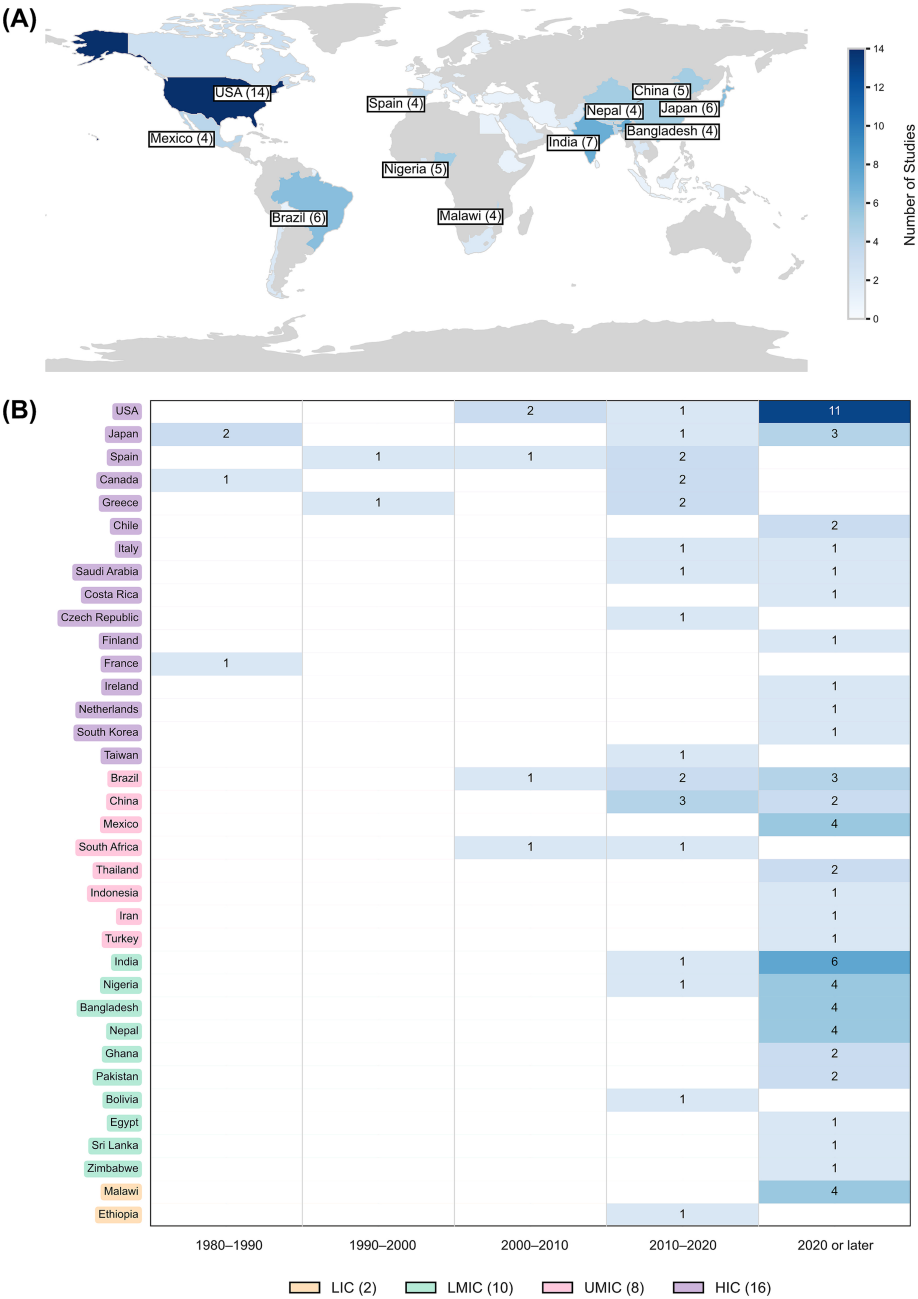
**(A)** Geographical distribution of countries reporting environmental surveillance of *Salmonella* spp. (*n* = 94); **(B)** Number of studies from different countries over the years 1980 to 2020 in 10-year intervals, and after 2020, along with the socioeconomic status. LIC: Low-income countries, LMIC: low- and middle-income countries, UMIC: upper middle-income countries, and HIC: high-income countries.

The selected studies represented a wide geographic distribution across 36 countries, five of them in SEAR countries (Figure 2A). Most (*n* = 89) were single-country studies, while five involved multiple countries. According to the World Bank’s income classification, the studies spanned two LICs, ten LMICs, eight UMICs, and 16 high-income countries (HICs). The United States of America and India contributed the highest number of studies published after 2020 (Figure 2B).

### 3.3 Reported laboratory methods

The included studies employed a range of sampling methods to collect wastewater, each reflecting different operational contexts and surveillance goals (Table 1). Most studies reported using grab sampling (*n* = 62), which involves collecting a predefined volume of wastewater in a sterile container at a single point in time. This was followed by trap sampling (*n* = 10), a passive technique in which a receptacle, often a Moore swab, is exposed to flowing wastewater over a set period to capture microorganisms. Composite sampling (*n* = 9) was also employed, involving autosamplers that collect wastewater at regular intervals over an extended period. Additionally, some studies (*n* = 7) employed a combination of grab and trap sampling, while five studies did not specify the type of sample collected. One study uniquely used sewage sludge as the sample type.

Sample handling after collection was reported in 61 studies (Table 1). Among these, 56 studies described the transportation of samples under cold chain conditions to preserve microbial integrity. Additionally, 52 studies provided details on the time elapsed between sample collection and laboratory processing. Of these, 49 studies processed samples within 24 h, while three studies reported delays exceeding 24 h.

Sample processing, defined as the steps taken before initiating the microbiological procedure, was also mentioned by various methods (*n* = 74) (Table 1). The methods reported processing using filtration (*n* = 32) to trap microorganisms or remove large debris, centrifugation (*n* = 8) to pellet the microorganisms, dilution or serial dilution (*n* = 5) to reduce inhibitory components, processing of the Moore swab to extract its contents (*n* = 3), using specially designed magnetic particles that bind with bacteria (*n* = 1), or a combination of methods (*n* = 23) to remove debris and inhibitors and trap microorganisms.

**Figure 3:**
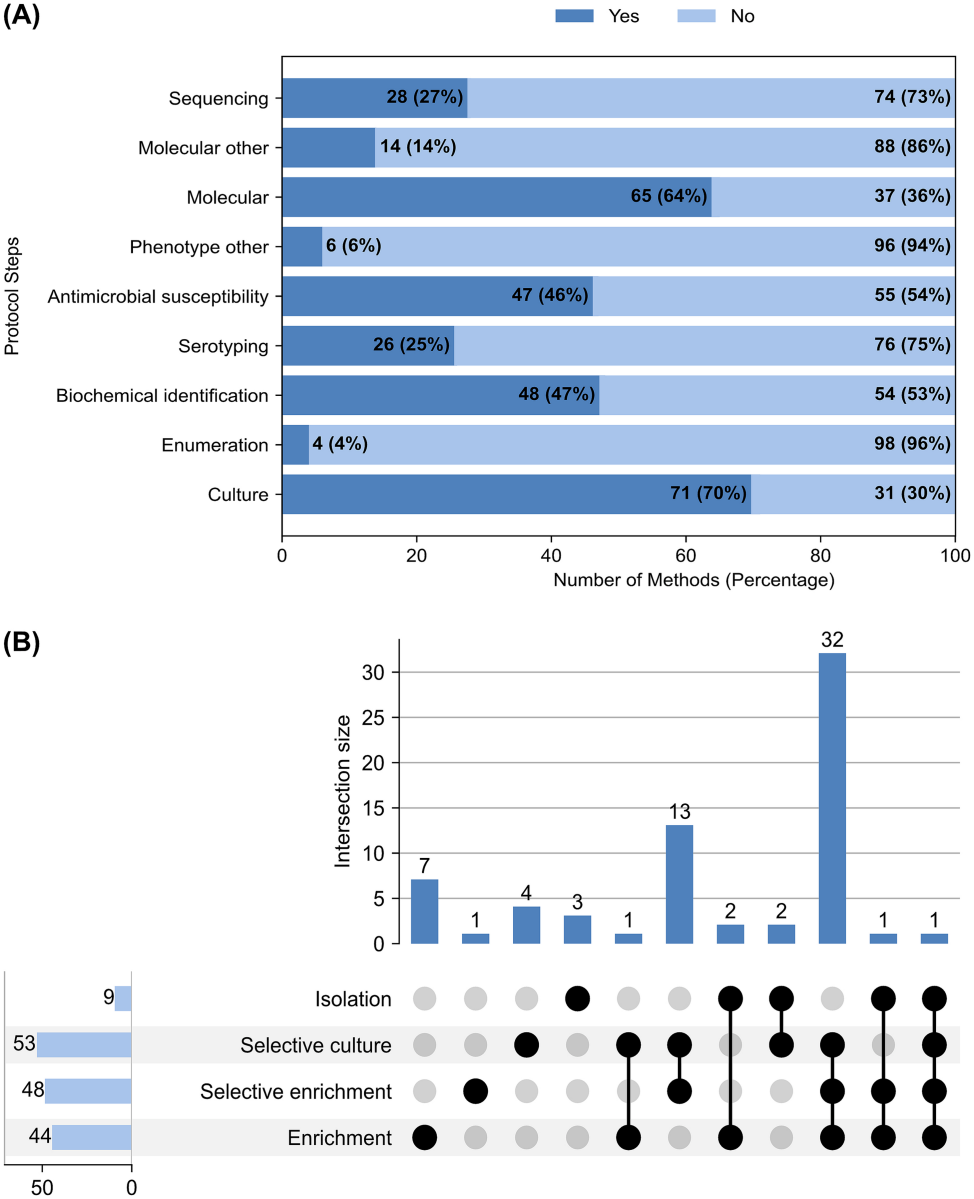
**(A)** Count of steps used by each method (*n* = 102); **(B)** Upset plot for culture steps used in the methods, showing intersection size, that is, the count of methods using culture steps either alone or in combination.

The laboratory testing could be further resolved in different protocol steps, including bacterial culture, bacterial enumeration, phenotypic characterization of observable bacterial traits (biochemical identification, serotyping, antimicrobial susceptibility, and other methods), genotypic characterization based on bacterial nucleic acid (PCR and other methods), and sequencing (Figure 3A). Two studies used a novel bacteriophage-based method that employed detecting *Salmonella*-specific bacteriophages as an indicator for *Salmonella*. It was noted that the use of different protocol steps is influenced by the central question or hypothesis that these studies aimed to address.

Bacterial culture was attempted by 70% of the methods (*n* = 71), as shown in Figure 3A. This step typically involved enrichment in non-selective or selective media, followed by selective culture or isolation of *Salmonella* spp., using standard culture media. However, not all studies included a culture step as part of their methodology, some relied solely on molecular or alternative detection techniques. Figure 3B illustrates the combination of culture steps used in different methods (*n* = 67), excluding four methods that reported using standard methods (ISO 6579, ISO 19250, FDA Bioanalytical manual protocol for *Salmonella*, and APHA standard method) and one method that exclusively used bacteriophage-specific methods. Among those that performed culture, the most common protocol involved enrichment, selective enrichment, and selective culture (*n* = 32), followed by selective enrichment and culture (*n* = 13). Additionally, four methods reported bacterial enumeration using serial dilutions and the most probable number (MPN) method to estimate bacterial load.

Figure 3A also illustrates how different phenotypic characterization assays were primarily used to characterize isolated bacteria based on observable traits, such as growth in specific media, serotype, or antimicrobial susceptibility. The phenotypic methods included biotyping with biochemical media (*n* = 48), serotyping (*n* = 26), antimicrobial susceptibility testing (*n* = 47), and other phenotypic techniques mainly involving phage typing (*n* = 3), Matrix-Assisted Laser Desorption/Ionization Time-of-Flight, MALDI-TOF (*n* = 2), and both MALDI-TOF and phage typing (*n* = 1). Biotyping utilized standard biochemical identification techniques, either manual (*n* = 32), automated (*n* = 12), or a combination of both (*n* = 3). One study did not specify the biochemicals used. For serotyping, most studies employed the Kauffman-White serotyping scheme to characterize *Salmonella* isolates (*n* = 24). Two studies reported the use of a PCR-based serotyping scheme. Regarding antimicrobial susceptibility, the majority of studies used the disc diffusion assay (*n* = 36), followed by broth microdilution (*n* = 4), automated systems (*n* = 3), a combination of disc diffusion and automated systems (*n* = 2), and a combination of disc diffusion and broth microdilution (*n* = 1). One of the studies also utilized resistance transfer testing to understand the mechanism of AMR gene transfer to a susceptible host.

Genotypic characterization of bacterial nucleic acid by molecular assays primarily included variants of PCR (*n* = 65), as well as other molecular assays, such as pulsed field gel electrophoresis (*n* = 10), plasmid analysis (*n* = 2), DNA fingerprinting (*n* = 1), fluorescence *in situ* hybridization (*n* = 1), and sequencing (*n* = 28). The methods reported included qPCR (*n* = 22), PCR (*n* = 18), molecular assays for antimicrobial resistance genes (ARG) (*n* = 12), and a combination of qPCR and ARG PCR (*n* = 2). Other than that, one study each reported using multiplex PCR, RT-PCR BioFire FilmArray® panel, crystal digital PCR, high-throughput qPCR, PCR with virulence marker PCR, culture PCR with high-throughput qPCR, 16S RNA PCR with PCR, qPCR with high-throughput qPCR, PCR with qPCR, and qPCR with denaturing gradient gel electrophoresis. Most used genomic method was whole genome sequencing, WGS (*n* = 11), followed by untargeted or shotgun metagenomics (*n* = 6), and 16S rDNA targeted sequencing (*n* = 2). One study each reported targeted sequencing, 16S rRNA sequencing, ARG genes sequencing, untargeted metagenomics, long and short read sequencing using Nanopore and Illumina sequencing, 16S rRNA sequencing with WGS, 16S RNA sequencing with metagenomics, and 16S rDNA sequencing with biomarker sequencing.

### 3.4 Identification of laboratory testing pathways

To further understand the commonalities in the reported methodologies of WES for *Salmonella* spp. In the selected papers an exploratory analysis was performed to identify major testing pathways from sample collection to testing. In this approach, 87 different methods from 79 studies were employed (Supplementary Table 10). Fifteen methods from 14 studies were excluded from this analysis because no sample was specified, standard procedures were not discussed in detail, bacteriophage surveillance was not included, or methods used for stored isolates were not specified.

**Figure 4:**
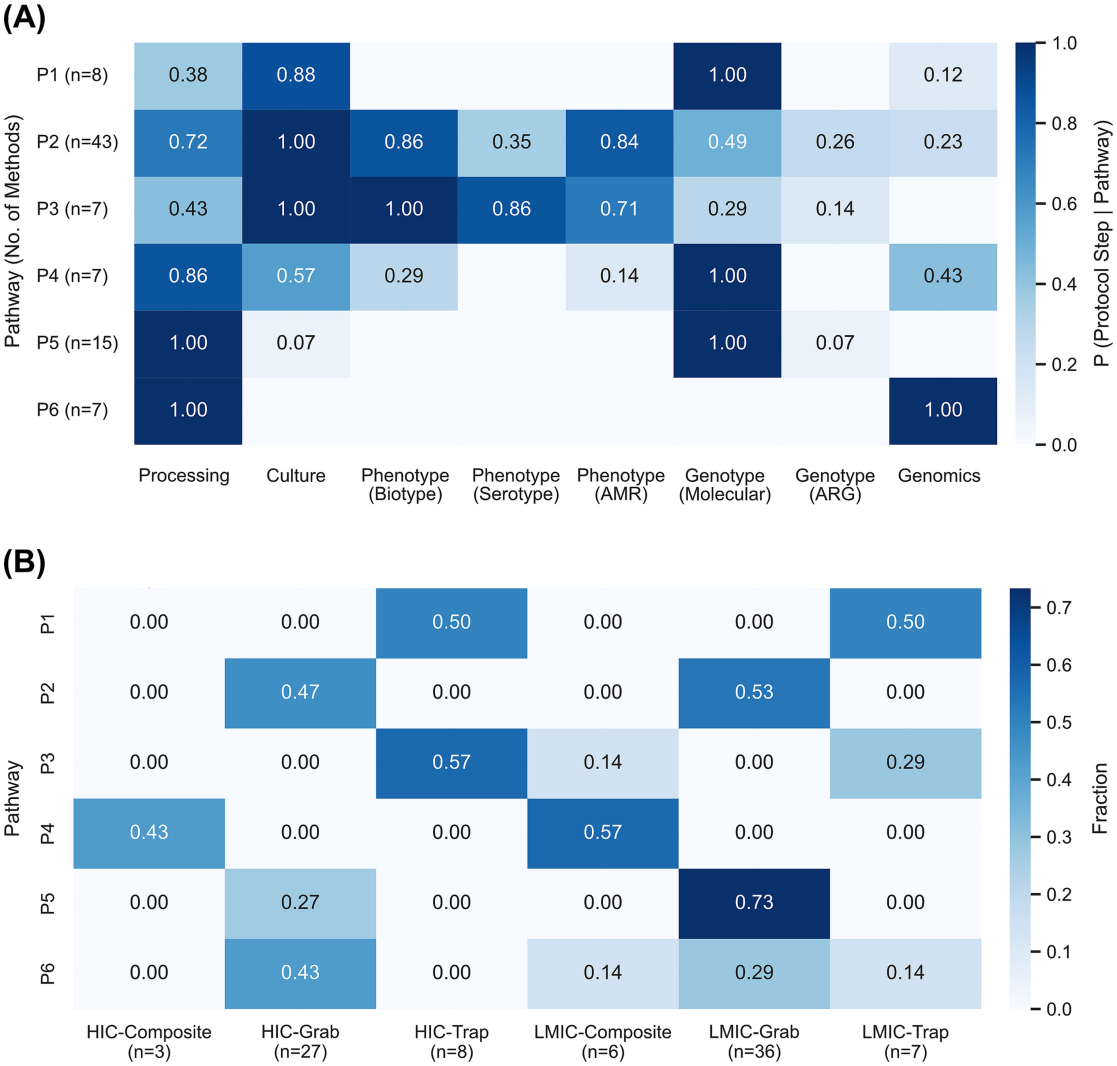
**(A)** Clustering of methods used to identify the pathways involved in wastewater sample processing and testing, based on the findings from 79 extracted studies that included 87 different methods. For each pathway cluster, the number of methods is indicated; **(B)** Fraction plot showing the use of pathways for testing by sample type and country category (LMIC group includes LIC, LMIC, and UMIC).

Based on the clustering, a total of six pathways were identified (Figure 4A; Supplementary Figure 3). The details of each method mapped to a pathway are provided in Supplementary Table 10. Eight methods shared the pathway P1, mainly involving a culture step followed by identification using molecular methods, with a slight association with a processing step. Pathway P2 was the most used pathway, with 43 methods. P2 has a strong association with the processing step, culture, biotyping of isolates, and antimicrobial susceptibility testing. The pathway was mildly associated with molecular assays, with low association to other steps. P3 was shared by seven methods and was associated with culture, biotyping, serotyping, and AST. Seven studies shared pathway P4, which was strongly associated with a processing step and the use of molecular assays for characterization. P4 also had a moderate association with culture and genomics methods. Pathway P5 was the second most used pathway, with 15 methods, primarily involving molecular characterization after a processing step. Lastly, pathway P6 was shared by seven methods and included a processing step and testing using a genomics method. Figure 4B illustrates the fractional distribution of methodological pathways across sample types in LMIC and HIC settings. Both LMICs and HICs studies commonly employed pathways P2, P5, and P6 for grab samples. Pathway P2 was used at similar rates in both income groups, while P5 was more prevalent in LMICs and P6 in HICs settings. In LMICs, P5 was also applied to composite and trap samples. For trap sampling, pathway P1 was used equally by both LMIC and HIC studies. Pathway P3 was preferred in HICs, whereas LMICs applied it to both trap and composite samples. Pathway P4 was predominantly associated with composite sampling across studies. Stability analysis of pathways confirmed that the identified pathways represent methodological choices by the authors. The exclusion of unclassified methods showed significantly lower reporting completeness score compared to the clustered pathways (*p* = 0.02) confirming filtering out these methods from the pathway clustering due to insufficient reported details. For the clustered methods, reporting scores were uniform with no difference in completeness scores detected by ANOVA (*p* = 0.17). To control for potential pseudoreplication, we performed a sensitivity analysis by aggregating completeness score at study level (*n* = 94), which yielded consistent result (p = 0.17). A stricter sensitivity analysis excluding removing papers with multiple methods was ruled out as it resulted in dropping of Pathway P1 sample size below the threshold required for reliable variance estimation (n = 3). Bootstrap analysis further demonstrated high internal stability, with CV remaining below 0.08 for all pathways (Supplementary Table 12). These results, although exploratory, indicate that the methodologies utilized for *Salmonella* WES can be generalized at the level of protocol steps, thereby providing an opportunity to identify critical steps and define quality control protocols for *Salmonella* WES.

### 3.5 Domain mapping for selected studies

**Figure 5:**
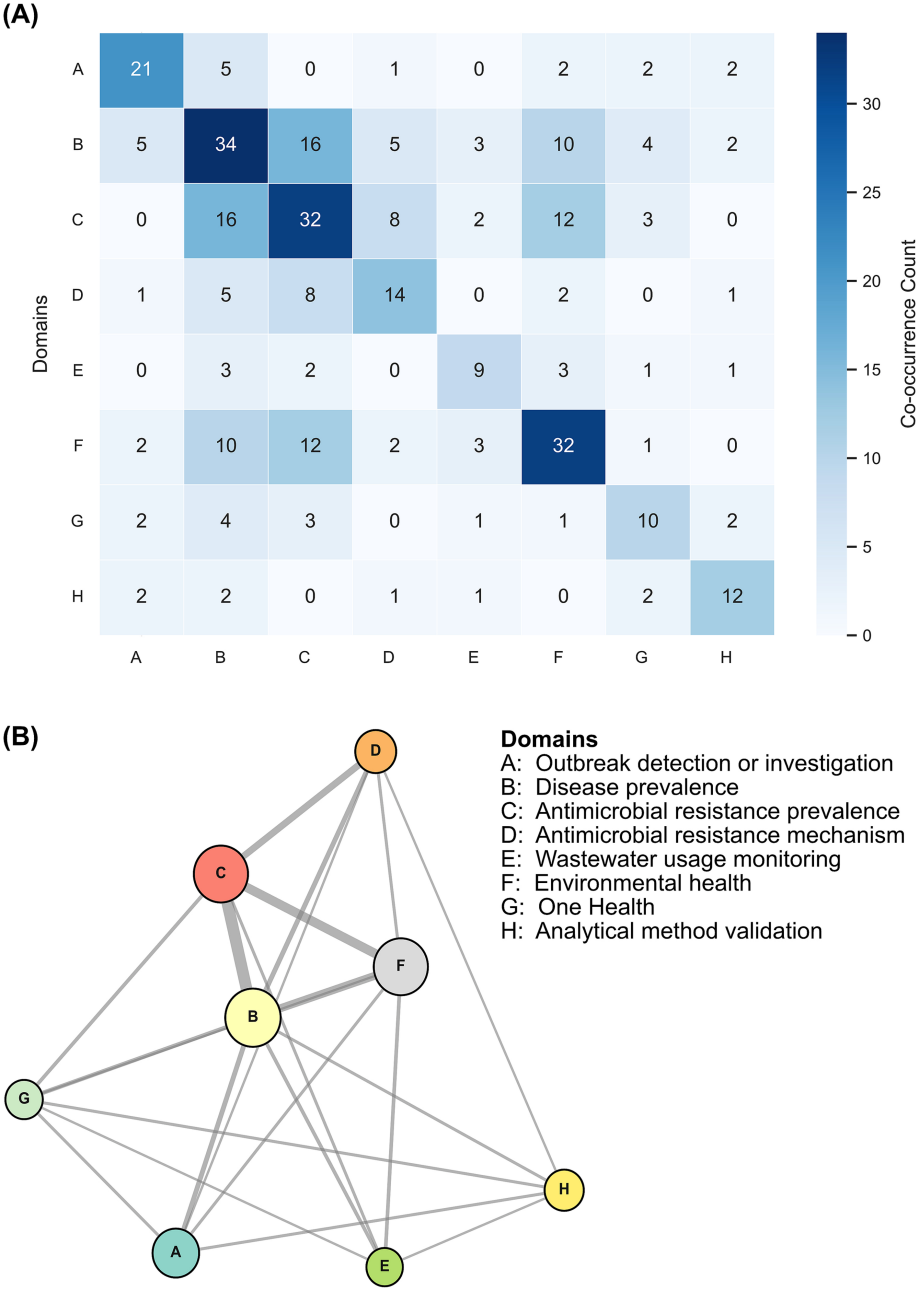
**(A)** Study domain co-occurrence map (*n* = 94); **(B)** Network of co-occurrence map of thematic domains (*n* = 94).

To further understand the thematic domains of the studies to provide context for planning of *Salmonella* WES, an exploratory analysis was done to assign thematic domainsto all 94 unique studies included in the systematic review (Figure 5A). Two authors identified the thematic domains examining titles, keywords, and abstracts; where structured abstracts were unavailable, the first page was reviewed. The details of the identified study domains are provided in Supplementary Table 11. Each study could be mapped to one or more domains. A total of eight domains were identified: Domain A - Outbreak detection and investigation (*n* = 33): studies using WES to detect, investigate, or retrospectively link outbreaks. Domain B - Disease prevalence (*n* = 79): studies using WES to supplement clinical or sentinel surveillance for estimating disease burden. Domain C - AMR prevalence (*n* = 73): studies assessing the spread of AMR organisms or genes in the community. Domain D - Mechanisms of AMR (*n* = 31): studies exploring the physiological, molecular, or genetic mechanisms of transmission of resistance via wastewater. Domain E - Wastewater usage monitoring (*n* = 19): studies evaluating microbial diversity in reclaimed or contaminated water used for agriculture or irrigation. Domain F - Environmental health (*n* = 62): studies investigating links between wastewater microbial diversity and anthropogenic or environmental factors. Domain G - One Health (*n* = 23): studies addressing human-animal-environment interactions, including cross-species transmission and intersectoral AMR evidence. Domain H - Method validation (*n* = 20): studies focused on validating WES methods for sample collection or testing, including assessment of assay sensitivity and standardization of protocols.

The co-occurrence matrix of the identified domains was mapped onto a network to understand relatedness between the identified thematic domains from the dataset of 94 included studies (Figure 5B). The network analysis revealed that domains B (Disease Prevalence), C (AMR Prevalence), and F (Environmental Health) are closely grouped. These may be considered as core domains that guide key questions in the field, such as how WES can detect the presence or absence of *Salmonella* spp., assess AMR, and understand the role of environmental factors in pathogen establishment. Domains D (Mechanisms of AMR) and E (Wastewater Usage Monitoring) may act as bridge or support domains, connecting strongly with the core domains and providing important context, such as understanding resistance mechanisms or identifying sources of contamination through wastewater reuse. Domains G (One Health) and H (Method Validation) may be linked to multiple domains but in smaller numbers, indicating their cross-cutting nature. These domains may contribute to broader perspectives, such as intersectoral collaboration or methodological rigor. Finally, domain A (Outbreak Detection and Investigation) is relatively isolated, with fewer connections to other domains. This suggests that studies in this domain may often require specialized approaches and may not overlap extensively with broader surveillance objectives. The identified domains can be used to provide important context to the *Salmonella* WES, especially in cases where public health context is not sufficiently provided by *Salmonella* Typhi.

### 3.6 Completeness of reported methods

To evaluate the completeness of reporting on the methods, a structured template (Supplementary Table T9) was used to determine whether the studies thoroughly documented the wastewater methodology. The extracted data is provided in the Supplementary material 2. Briefly, the manuscripts were reviewed by two authors and graded as per the rubric outlined in Supplementary Table T9. All the disagreements were resolved by consensus between all authors. The methodological quality assessment criteria also classified the studies based on the study methodology (Figure 1), however, this metric only classifies the studies based on completeness of the reporting of the methodology and not on the quality of the method. Supplementary Figure 4 illustrates how studies (*n* = 94) reported the methodology across various assessment criteria. Nearly all studies clearly provided information on sampling site details (*n* = 79), sample processing (*n* = 86), and testing methods (*n* = 79). Additionally, the choice of site, based on the hypothesis or central question posed by the study (*n* = 60), sample collection details (*n* = 60), details of testing procedures, including reagents (*n* = 53), and sample transport conditions with transient times (*n* = 46), were reported inconsistently. The reporting of quality control procedures used for laboratory methods remains the only criterion that is not frequently reported (n = 13). The completeness assessment of reported methodology highlighted that due to lack of widely accepted standardized methodology, most of the included studies documented aspects such as site selection, sample handling, transport, and testing with reasonable consistency, while under-reporting the quality control measures needed to ensure reproducibility of results.

### 3.7 Recommendations on protocols and public health question framing

**Figure 6:**
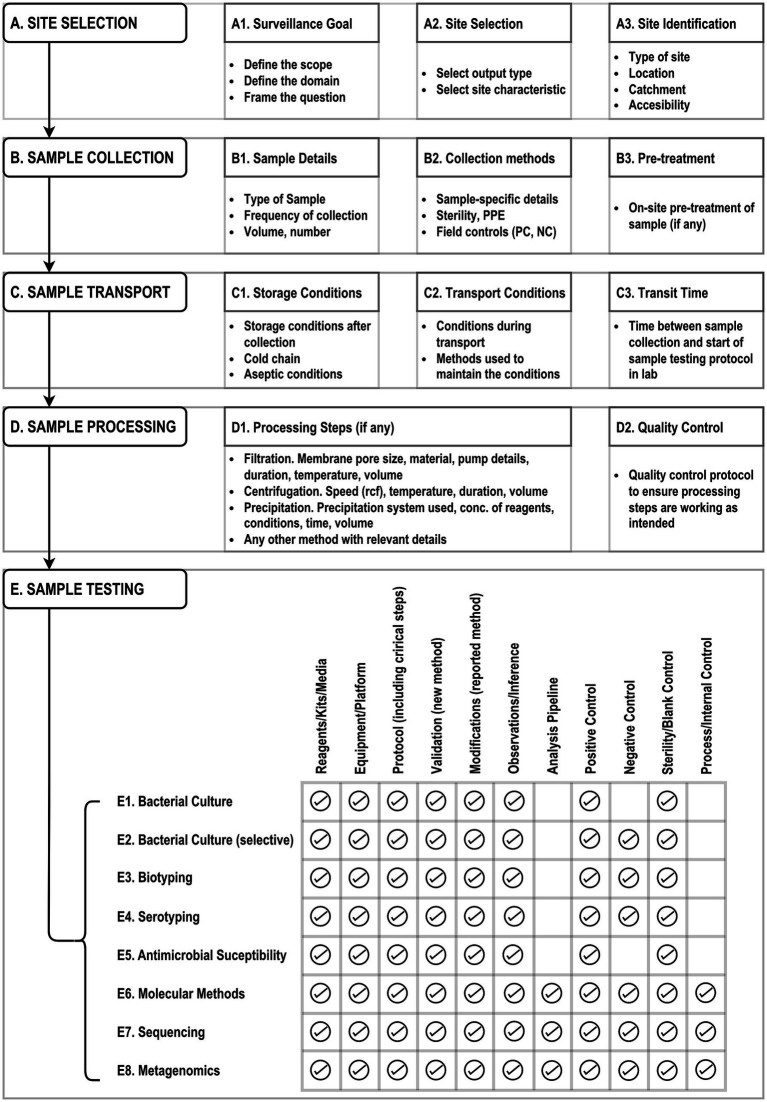
Recommendations for details to be included while reporting the WES methodologies for *Salmonella.*

In view of the lack of standardized methodology or guidance on *Salmonella* wastewater surveillance, we have attempted to outline recommendations that could support countries in initiating WES for *Salmonella* (Figure 6). Figure 6 provides detailed technical guidance on various steps that should be considered while implementing WES for *Salmonella*. It is also essential for the reproducibility of the selected method that all information for the chosen steps is included, either within the manuscript, Supplementary material, or as an online-published protocol during publication.

The knowledge gained in this systematic review is synthesized into a heuristic framework for resource planning to assist all stakeholders, including public health program managers, laboratory directors, donors, and policy makers, in framing the right questions (Table 2). The developed framework provides essential context for defining the public health goals of establishing WES for *Salmonella*, including its scope and domain. The core domains may help shape the main questions, while the supporting domains add additional objectives may strengthen the impact of the central questions. Cross-cutting domains can potentially provide context when multi-sectoral engagement is necessary. Niche domains may be used in conjunction with other domains to frame questions but will often require specific objectives that may not be relevant to different sectors. The framed question then guides the selection of suitable output data and sites to meet the goals, as well as justifying the testing pathway based on the available resources and infrastructure. The chosen testing pathway can be made reproducible by following the recommendations in Figure 6. Therefore, the proposed framework serves as a foundational evidence-based instrument for resource planning while simultaneously facilitating consensus building for among various stakeholders for initiating *Salmonella* WES.

**Table 2:** Decision tool for developing a reason-based WES program in resource-limited settings.

Public health scope	Domains (Type)	Output type (Site selection)	Testing pathways
Monitoring for the detection of importations Monitoring of community-level baselines Monitoring of changes in risk factors Monitoring of epidemiological changes Monitoring the effect of changes in healthcare practices Optimization of resource and budget allocation Evidence generation for developing and implementing public health policies Evidence generation for evaluation of public health policies Evidence generation for calibration of public health interventions	Outbreak detection or identification (Niche) Disease Prevalence (Core) AMR Prevalence (Core) and/or Mechanisms (Supporting) Monitoring wastewater usage (Supporting) Environmental Health (Core) One Health (Cross-cutting) Analytical Method Validation (Cross-cutting)	Cross-sectional (single site, single time) Time-series (single site, multiple times) Longitudinal (multiple sites, multiple times) Spatial (multiple sites) Spatio-temporal (multiple sites, multiple times) Hierarchical (nested structure of sites, e.g., small to large drains) Network (interconnected site, e.g., multiple drains linked to WWTP)	P1 P2 P3 P4 P5 P6

## 4 Discussion

### 4.1 Summary of key findings

This systematic review identified substantial heterogeneity in laboratory methodologies used for WES of *Salmonella* spp. across 94 studies spanning 36 countries. A total of 102 distinct methodological approaches were documented, reflecting wide variation in sampling strategies, sample types, and laboratory testing protocols. Grab sampling was the most common method, although trap and composite sampling were also used, often without consistent reporting on sample handling or transport conditions. Culture-based methods were frequently employed, yet many studies relied solely on molecular or genomic techniques, underscoring the lack of standardized testing pathways. Six distinct methodological pathways were identified, each reflecting different combinations of protocol steps and resource contexts.

Domain mapping showed that studies often addressed multiple public health objectives, with disease prevalence, AMR, and environmental health emerging as core domains. Outbreak detection and method validation were underrepresented, suggesting a need for targeted investment in these areas. The interdisciplinary nature of *Salmonella* WES, encompassing One Health, environmental monitoring, and epidemiology, reflects its evolving role in integrated public health action (127, 128). The identified domains emphasize the importance of *Salmonella* WES in providing community-level signals for disease prevalence, information on pathogen importation, characterization of AMR emergence and spread, and assessment of environmental transmission risk, which directly align with the WHO-defined potential use cases for routine WES (27).

### 4.2 Context for interpretation of results

The findings must be interpreted in the context of infrastructural and epidemiological realities in LMICs, particularly in the WHO South-East Asia Region. Rapid urbanization has outpaced the development of sanitation infrastructure, resulting in fragmented wastewater systems that complicate the recovery and surveillance of pathogens (129, 130). Wastewater reuse for agriculture and urban needs has increased exposure to waterborne diseases, including typhoid fever (131–137).

Historically, *Salmonella* Typhi was among the first pathogens monitored in sewage to identify asymptomatic carriers (138), and this approach has been used to locate transmission hotspots (139). Despite its early promise, WES for typhoid has remained underutilized, with poliovirus being the only pathogen for which environmental surveillance is widely institutionalized (2–4, 140). The infrastructure established for wastewater sample collection and molecular testing for polio ES could be leveraged to initiate *Salmonella* WES. The COVID-19 pandemic catalyzed renewed interest in wastewater-based surveillance, demonstrating its utility for early detection and public health decision-making (140, 141).

The post-pandemic surge in publications reflects this shift, with studies emerging from both LMICs and HICs. Unconventional sampling sources; such as aircraft, refugee ships, and border entry points, have expanded the scope of surveillance (142–145). However, the dominance of grab sampling, limited use of Moore swabs (146), and inconsistent reporting of sample handling suggest that feasibility often outweighs methodological rigor.

### 4.3 Implications of results in the WES domain

The lack of standardization has direct implications for reproducibility and comparability. Our quality assessment revealed that while most studies reported basic methodological components, critical details such as quality control procedures, reagent specifications, and validation criteria were frequently omitted (139, 147–150). This gap limits the utility of published protocols for replication or scale-up in other settings.

To address this, we categorized the methods into six distinct pathways based on protocol steps and resource contexts. This classification offers a pragmatic framework for selecting appropriate methodologies aligned with laboratory capacity and surveillance goals. Importantly, the heuristic framework for resource planning developed from this synthesis (Table 2) enables stakeholders to align methodological choices with public health objectives, whether for outbreak detection, disease burden estimation, or AMR monitoring.

The interdisciplinary nature of *Salmonella* WES calls for integrated policy frameworks. Surveillance programs should be embedded within broader public health strategies that facilitate cross-sectoral collaboration among human, animal, and environmental health agencies (127, 128). This aligns with the Quadripartite One Health Joint Plan of Action and supports the development of multisectoral early warning systems for emerging pathogens (151).

### 4.4 Limitations of the study

Despite efforts to optimize the search strategy, the review may have missed relevant studies published in non-indexed journals or in languages other than English. The reliance on published literature means that methodological details were often incomplete or inconsistently reported, particularly regarding quality control procedures, reagent specifications, and validation criteria. This limited the ability to fully assess reproducibility and operational feasibility. The review also did not include grey literature, internal reports, or unpublished protocols, which may contain valuable insights into real-world implementation challenges. While the heuristic framework for resource planning, thematic domain identification, and pathway classification are grounded in extracted data, they have not yet been validated through field testing or stakeholder consultation, which we aim to do as a next step. The review focused exclusively on wastewater or contaminated surface waters sources and excluded other environmental matrices such as surface waters, which may also be relevant for *Salmonella* surveillance in certain contexts such as one health.

### 4.5 Recommendations and future directions

To advance WES for typhoid control, we recommend the development and adoption of standardized protocols that are both scientifically robust and operationally feasible across varied infrastructure settings. These protocols should include clear specifications for sample collection (e.g., volume, timing, and type), transport conditions, and laboratory testing workflows along with validation criteria for result interpretation. The consistent use of Moore swabs, which have demonstrated superior sensitivity in flowing wastewater environments, should be encouraged in typhoid-endemic regions (146). Furthermore, quality control procedures must be embedded throughout the surveillance process, with explicit documentation of reagents, test conditions, and validation criteria to ensure methodological transparency and reliability (139, 147–150).

Surveillance systems must be tailored to local resource contexts. The six methodological pathways identified in this review provide a flexible framework for laboratories with differing capacities. These pathways can be matched to surveillance objectives using the heuristic framework developed herein, which links public health goals to appropriate testing strategies and site selection models. Such alignment is particularly critical in LMICs, where infrastructure constraints necessitate pragmatic and cost-effective approaches.

Strategically, *Salmonella* WES should be integrated into national disease control programs and linked to typhoid conjugate vaccine (TCV) deployment. Environmental data can complement clinical surveillance and inform the timing and targeting of vaccination campaigns, especially in settings where blood culture-based diagnostics are limited (27, 152–154).

Future studies should evaluate the sensitivity and cost-effectiveness of composite sampling approaches, particularly in decentralized and low-flow wastewater systems common in LMICs. Comparative assessments of the six identified methodological pathways under field conditions are needed to determine which combinations of protocol steps yield the most reliable results across diverse environmental contexts.

Additionally, research should explore the integration of WES data with clinical and AMR surveillance systems to enhance early warning capabilities and inform vaccine deployment strategies. The operational feasibility of implementing surveillance in unconventional settings; such as border crossings, refugee camps, and transportation hubs, also requires further study, especially in light of emerging global health threats.

Finally, interdisciplinary implementation models that align with One Health frameworks should be piloted and evaluated. These models must address not only technical performance but also governance, stakeholder engagement, and sustainability in resource-limited settings.

## 5 Conclusion

WES for *Salmonella* spp., particularly *Salmonella* Typhi, presents a promising yet underutilized tool for public health action in resource-limited settings. Standardized protocol and its harmonized implementation are thus a key success factor for *Salmonella* WES, however, it necessitates scientific and operational research to achieve the outcome. Leveraging existing systems for polio ES has potential to expedite the implementation of *Salmonella* WES. This review offers a foundation for methodological harmonization, strategic integration, and interdisciplinary collaboration. By aligning surveillance design with public health objectives and local capacities, stakeholders can advance robust, scalable systems that support typhoid control and broader One Health goals.

## Data Availability

The original contributions presented in the study are included in the article/Supplementary material, further inquiries can be directed to the corresponding author.

## References

[1] GPEI. Global polio eradication initiative Geneva, Switzerland: World Health Organization (2025) Available online at: https://polioeradication.org/.

[2] World Health Organization. Guidelines for environmental surveillance of poliovirus circulation. Geneva, Switzerland: World Health Organization, Department of Vaccines and Biologicals (2003).

[3] GPEI. Guidelines on environmental surveillance for detection of poliovirus. Guideline. Geneva: Global Polio Eradication Initiative, World Health Organization, Initiative. GPE (2015).

[4] GPEI. Field guidance for the implementation of environmental surveillance for poliovirus. Geneva: Global Polio Eradication Initiative, World Health Organization (2023).

[5] Levy JI Andersen KG Knight R Karthikeyan S. Wastewater surveillance for public health. Science. (2023) 379:26–7. doi: 10.1126/science.ade2503, 36603089 PMC10065025

[6] Grassly NC Shaw AG Owusu M. Global wastewater surveillance for pathogens with pandemic potential: opportunities and challenges. Lancet Microbe. (2025) 6:100939. doi: 10.1016/j.lanmic.2024.07.002, 39222653

[7] Tiwari A Radu E Kreuzinger N Ahmed W Pitkänen T. Key considerations for pathogen surveillance in wastewater. Sci Total Environ. (2024) 945:173862. doi: 10.1016/j.scitotenv.2024.173862, 38876348

[8] Toro L de Valk H Zanetti L Huot C Tarantola A Fournet N . Pathogen prioritisation for wastewater surveillance ahead of the Paris 2024 Olympic and Paralympic games, France. Euro Surveill. (2024) 29:2400231. doi: 10.2807/1560-7917.ES.2024.29.28.2400231, 38994605 PMC11241851

[9] World Health Organization In: World Health Organization, editor. Wastewater and environmental surveillance for one or more pathogens – Guidance on prioritization, implementation and integration. Geneva: World Health Organization (2024).

[10] Wang H Zhang P Zhao Q Ma W. Global burden, trends and inequalities for typhoid and paratyphoid fever among children younger than 15 years over the past 30 years. J Travel Med. (2024) 31:taae140. doi: 10.1093/jtm/taae140, 39450993

[11] Kim S Lee KS Pak GD Excler J-L Sahastrabuddhe S Marks F . Spatial and temporal patterns of typhoid and paratyphoid fever outbreaks: a worldwide review, 1990–2018. Clin Infect Dis. (2019) 69:S499–509. doi: 10.1093/cid/ciz705, 31665782 PMC6821269

[12] Ferrari RG Rosario DKA Cunha-Neto A Mano SB Figueiredo EES Conte-Junior CA. Worldwide epidemiology of *Salmonella* serovars in animal-based foods: a meta-analysis. Appl Environ Microbiol. (2019) 85:e00591–19. doi: 10.1128/aem.00591-19, 31053586 PMC6606869

[13] Hoelzer K Moreno Switt AI Wiedmann M. Animal contact as a source of human non-typhoidal salmonellosis. Vet Res. (2011) 42:34. doi: 10.1186/1297-9716-42-34, 21324103 PMC3052180

[14] Lu X Luo M Wang M Zhou Z Xu J Li Z . High carriage and possible hidden spread of multidrug-resistant Salmonella among asymptomatic Workers in Yulin, China. Nat Commun. (2024) 15:10238. doi: 10.1038/s41467-024-54405-9, 39592576 PMC11599845

[15] Khanam F Darton TC Meiring JE Kumer Sarker P Kumar Biswas P Bhuiyan MAI . *Salmonella Typhi* stool shedding by patients with enteric fever and asymptomatic chronic carriers in an endemic urban setting. J Infect Dis. (2021) 224:S759–63. doi: 10.1093/infdis/jiab476, 34586391 PMC8687075

[16] Uwanibe JN Kayode TA Oluniyi PE Akano K Olawoye IB Ugwu CA . The prevalence of undiagnosed *Salmonella enterica* serovar Typhi in healthy school-aged children in Osun state, Nigeria. Pathogens. (2023) 12:594. doi: 10.3390/pathogens12040594, 37111480 PMC10140884

[17] Cao Y Karthikeyan AS Ramanujam K Raju R Krishna S Kumar D . Geographic pattern of typhoid fever in India: a model-based estimate of cohort and surveillance data. J Infect Dis. (2021) 224:S475–83. doi: 10.1093/infdis/jiab187, 35238365 PMC8892532

[18] Mawazo A Bwire GM Matee MIN. Performance of Widal test and stool culture in the diagnosis of typhoid fever among suspected patients in Dar Es Salaam, Tanzania. BMC Res Notes. (2019) 12:316. doi: 10.1186/s13104-019-4340-y, 31167646 PMC6551910

[19] Andualem G Abebe T Kebede N Gebre-Selassie S Mihret A Alemayehu H. A comparative study of Widal test with blood culture in the diagnosis of typhoid fever in febrile patients. BMC Res Notes. (2014) 7:653. doi: 10.1186/1756-0500-7-653, 25231649 PMC4177418

[20] Aiemjoy K Seidman JC Saha S Munira SJ Islam Sajib MS Sium SMA . Estimating typhoid incidence from community-based Serosurveys: a multicohort study. The Lancet Microbe. (2022) 3:e578–87. doi: 10.1016/S2666-5247(22)00114-8, 35750069 PMC9329131

[21] Kumar S Nodoushani A Khanam F DeCruz AT Lambotte P Scott R . Evaluation of a rapid point-of-care multiplex immunochromatographic assay for the diagnosis of enteric fever. mSphere. (2020) 5:e00253. doi: 10.1128/msphere.00253-20 32522777 PMC7289704

[22] Uzzell CB Abraham D Rigby J Troman CM Nair S Elviss N . Environmental surveillance for Salmonella Typhi and its association with typhoid fever incidence in India and Malawi. J Infect Dis. (2024) 229:979–87. doi: 10.1093/infdis/jiad427, 37775091 PMC11011185

[23] Yanagimoto K Yamagami T Uematsu K Haramoto E. Characterization of Salmonella isolates from wastewater treatment plant influents to estimate unreported cases and infection sources of salmonellosis. Pathogens. (2020) 9:52. doi: 10.3390/pathogens9010052, 31936747 PMC7168602

[24] Abraham D Kathiresan L Sasikumar M Aiemjoy K Charles RC Kumar D . Wastewater surveillance for *Salmonella* Typhi and its association with seroincidence of enteric fever in Vellore, India. PLoS Negl Trop Dis. (2025) 19:e0012373. doi: 10.1371/journal.pntd.0012373, 40029872 PMC11896026

[25] Diemert S Yan T. Municipal wastewater surveillance revealed a high community disease burden of a rarely reported and possibly subclinical *Salmonella enterica* Serovar Derby strain. Appl Environ Microbiol. (2020) 86:e00814–20. doi: 10.1128/AEM.00814-20, 32591375 PMC7440783

[26] Yan T O'Brien P Shelton JM Whelen AC Pagaling E. Municipal wastewater as a microbial surveillance platform for enteric diseases: a case study for Salmonella and salmonellosis. Environ Sci Technol. (2018) 52:4869–77. doi: 10.1021/acs.est.8b00163, 29630348

[27] World Health Organization. Wastewater and environmental surveillance: Summary for typhoid and paratyphoid. Geneva: World Health Organization, Water S, Hygiene and Health (WSH) (2024).

[28] Jahan F Nasim MI Wang Y Kamrul Bashar SM Hasan R Suchana AJ . Integrating wastewater surveillance and meteorological data to monitor seasonal variability of enteric and respiratory pathogens for infectious disease control in Dhaka City. Int J Hyg Environ Health. (2025) 267:114591. doi: 10.1016/j.ijheh.2025.114591, 40403455

[29] Oktaria V Murni IK Handley A Donato CM Nuryastuti T Supriyati E . Environmental surveillance for *Salmonella Typhi* to detect the typhoid burden in Yogyakarta, Indonesia. Int J Hyg Environ Health. (2025) 266:114572. doi: 10.1016/j.ijheh.2025.114572, 40163994 PMC12042821

[30] Sotelo TJ Satoh H Mino T. Assessing wastewater management in the developing countries of Southeast Asia: underlining flexibility in appropriateness. J Water Environ Technol. (2019) 17:287–301. doi: 10.2965/jwet.19-006

[31] Nasim N El-Zein A Thomas J. A review of rural and Peri-urban sanitation infrastructure in South-East Asia and the Western Pacific: highlighting regional inequalities and limited data. Int J Hyg Environ Health. (2022) 244:113992. doi: 10.1016/j.ijheh.2022.113992, 35752101

[32] Owusu M Darko E Akortia D Nkrumah G Twumasi-Ankrah S Owusu-Ansah M . Evaluation of Moore and Grab sampling method for *Salmonella Typhi* detection in environmental samples in Ghana. PLoS One. (2025) 20:e0318840. doi: 10.1371/journal.pone.0318840, 40009631 PMC11864537

[33] Page MJ McKenzie JE Bossuyt PM Boutron I Hoffmann TC Mulrow CD . The Prisma 2020 statement: an updated guideline for reporting systematic reviews. BMJ. (2021) 372:n71. doi: 10.1136/bmj.n71, 33782057 PMC8005924

[34] Bramer WM Rethlefsen ML Kleijnen J Franco OH. Optimal database combinations for literature searches in systematic reviews: a prospective exploratory study. Syst Rev. (2017) 6:245. doi: 10.1186/s13643-017-0644-y, 29208034 PMC5718002

[35] Bramer WM. Variation in number of hits for complex searches in Google scholar. J Med Libr Assoc. (2016) 104:143–5. doi: 10.3163/1536-5050.104.2.009, 27076802 PMC4816485

[36] Gusenbauer M Haddaway NR. Which academic search systems are suitable for systematic reviews or Meta-analyses? Evaluating retrieval qualities of Google scholar, Pubmed, and 26 other resources. Res Synth Methods. (2020) 11:181–217. Epub 2019/10/16. doi: 10.1002/jrsm.1378, 31614060 PMC7079055

[37] Fantini D. Easypubmed: Search and retrieve scientific publication records from Pubmed. R package version 2.13, 2019 2019

[38] Grames EM Stillman AN Tingley MW Elphick CS. An automated approach to identifying search terms for systematic reviews using keyword co-occurrence networks. Methods Ecol Evol. (2019) 10:1645–54. doi: 10.1111/2041-210X.13268

[39] Kung J. Polyglot search translator. J Can Health Libr Assoc. (2022) 43:35–9. doi: 10.29173/jchla29600

[40] Acheamfour CL Parveen S Hashem F Sharma M Gerdes ME May EB . Levels of *Salmonella enterica* and *Listeria monocytogenes* in alternative irrigation water vary based on water source on the eastern shore of Maryland. Microbiol Spectr. (2021) 9:11. doi: 10.1128/Spectrum.00669-21 PMC8510256 34612697

[41] Agbo O Momoh M Odimegwu D Adonu C. Colistin resistance in WHO-designated global priority pathogens isolated from wastewater effluents of two hospitals in Enugu Metropolis, south East Nigeria. Journal of Medical Microbiology and Infectious Diseases. (2024) 12:110–20. doi: 10.61186/JoMMID.12.2.110

[42] Allsing N Kelley ST Fox AN Sant KE. Metagenomic analysis of microbial contamination in the U.S. portion of the Tijuana River watershed. Int J Environ Res Public Health. (2023) 20:600. doi: 10.3390/ijerph20010600 PMC9819409 36612923

[43] Arvanitidou M Tsakris A Constantinidis TC Katsouyannopoulos VC. Transferable antibiotic resistance among *Salmonella* strains isolated from surface waters. Water Res. (1997) 31:1112–6. doi: 10.1016/S0043-1354(96)00340-5

[44] Ballesteros-Nova NE Sánchez S Steffani JL Sierra LC Chen Z Ruíz-López FA . Genomic epidemiology of *Salmonella Enterica* circulating in surface waters used in agriculture and aquaculture in Central Mexico. Appl Environ Microbiol. (2022) 88:e0214921. doi: 10.1128/aem.02149-21, 35020454 PMC8904062

[45] Bell JB Macrae WR Elliott GE. Incidence of R factors in coliform, fecal coliform, and Salmonella populations of the Red River in Canada. Appl Environ Microbiol. (1980) 40:486–91. doi: 10.1128/aem.40.3.486-491.1980, 6999991 PMC291610

[46] Berge ACB Dueger EL Sischo WM. Comparison of *Salmonella enterica* serovar distribution and antibiotic resistance patterns in wastewater at municipal water treatment plants in two California cities. J Appl Microbiol. (2006) 101:1309–16. doi: 10.1111/j.1365-2672.2006.03031.x, 17105561

[47] Cangola J Abagale FK Cobbina SJ Osei RA. Prevalence of antibiotic-resistant Enterobacteriaceae in domestic wastewater and associated health risks in reuse practices. Int J Hyg Environ Health. (2025) 263:114478. doi: 10.1016/j.ijheh.2024.114478, 39369488

[48] Ceballos BSO Soares NE Moraes MR Catão RMR Konig A. Microbiological aspects of an Urban River used for unrestricted irrigation in the semi-arid region of north-East Brazil. Water Sci Technol. (2003) 47:51–7. doi: 10.2166/wst.2003.0159, 12639005

[49] Chen Z Moreno-Switt AI Reyes-Jara A Suarez ED Adell AD Oliveira CJB . A multicenter genomic epidemiological investigation in Brazil, Chile, and Mexico reveals the diversity and persistence of *Salmonella* populations in surface waters. MBio. (2024) 15:e0077724. doi: 10.1128/mbio.00777-24 38920393 PMC11253603

[50] Cheung S Zhou NA Ruhanya V Jesser KJ Nezomba I Musvibe J . Characterization of enteric pathogens in Harare, Zimbabwe using environmental surveillance and metagenomics. J Water Health. (2025) 23:477–92. doi: 10.2166/wh.2025.333, 40298267

[51] Chigwechokha P Nyirenda RL Dalitsani D Namaumbo RL Kazembe Y Smith T . *Vibrio cholerae* and *Salmonella typhi* culture-based wastewater or non-sewered sanitation surveillance in a resource-limited region. J Expo Sci Environ Epidemiol. (2024) 34:432–9. doi: 10.1038/s41370-023-00632-z, 38177335

[52] Cho S Hiott LM House SL Woodley TA McMillan EA Sharma P . Analysis of *Salmonella enterica* isolated from a mixed-use watershed in Georgia, USA: antimicrobial resistance, serotype diversity, and genetic relatedness to human isolates. Appl Environ Microbiol. (2022) 88:e0039322. doi: 10.1128/aem.00393-22, 35532233 PMC9128517

[53] Cho S Hiott LM Read QD Damashek J Westrich J Edwards M . Distribution of antibiotic resistance in a mixed-use watershed and the impact of wastewater treatment plants on antibiotic resistance in surface water. Antibiotics. (2023) 12:1586. doi: 10.3390/antibiotics12111586, 37998788 PMC10668835

[54] Chukwu EE Okwuraiwe A Kunle-Ope CN Igbasi UT Onyejepu N Osuolale K . Surveillance of public health pathogens in Lagos wastewater canals: a cross-sectional study. BMC Public Health. (2024) 24:3590. doi: 10.1186/s12889-024-21157-6, 39725906 PMC11670414

[55] Cioffi B Ianiro G Iaccarino D D'Apice F Ferraro A Race M . A potential risk assessment tool to monitor pathogens circulation in coastal waters. Environ Res. (2021) 200:111748. doi: 10.1016/j.envres.2021.111748, 34303676

[56] Díaz-Palafox G Tamayo-Ordoñez YJ Bello-López JM Ayil-Gutiérrez BA RodrÍguez-Garza MM de la Antonio Rodríguez- Garza J . Regulation transcriptional of antibiotic resistance genes (Args) in bacteria isolated from Wwtp. Curr Microbiol. (2023) 80:338. doi: 10.1007/s00284-023-03449-z 37672120 PMC10482803

[57] Díaz-Torres O Lugo-Melchor OY de Anda J Gradilla-Hernández MS Amézquita-López BA Meza-Rodríguez D. Prevalence, distribution, and diversity ofsalmonellastrains isolated from a subtropical lake. Front Microbiol. (2020) 11:16. doi: 10.3389/fmicb.2020.521146, 33042046 PMC7518123

[58] Economou V Gousia P Kansouzidou A Sakkas H Karanis P Papadopoulou C. Prevalence, antimicrobial resistance and relation to indicator and pathogenic microorganisms of *Salmonella enterica* isolated from surface waters within an agricultural landscape. Int J Hyg Environ Health. (2013) 216:435–44. doi: 10.1016/j.ijheh.2012.07.004, 22901425

[59] El-Tayeb MA Ibrahim ASS Al-Salamah AA Almaary KS Elbadawi YB. Prevalence, serotyping and antimicrobials resistance mechanism of *Salmonella enterica* isolated from clinical and environmental samples in Saudi Arabia. Braz J Microbiol. (2017) 48:499–508. doi: 10.1016/j.bjm.2016.09.021, 28245965 PMC5498448

[60] Espigares E Bueno A Espigares M Gálvez R. Isolation of Salmonella serotypes in wastewater and effluent: effect of treatment and potential risk. Int J Hyg Environ Health. (2006) 209:103–7. doi: 10.1016/j.ijheh.2005.08.006, 16373208

[61] Fu S Zhang Y Wang R Deng Z He F Jiang X . Longitudinal wastewater surveillance of four key pathogens during an unprecedented large-scale Covid-19 outbreak in China facilitated a novel strategy for addressing public health priorities-a proof of concept study. Water Res. (2023) 247:120751. doi: 10.1016/j.watres.2023.120751, 37918201

[62] Goldblum ZS M'Ikanatha NM Nawrocki EM Cesari N Kovac J Dudley EG. *Salmonella* sp. tied to multistate outbreak isolated from wastewater, United States, 2022. Emerg Infect Dis. (2024) 30:2695–7. doi: 10.3201/eid3012.240443, 39592600 PMC11616661

[63] Guruge SK Han Z Karunaratne SHPP Chandrajith R Cooray T Hu C . Short- and long-read metagenomics uncover the mobile extended spectrum Β-lactamase (Esbl) and carbapenemase genes in hospital wastewater in Sri Lanka. Water Res. (2025) 283:123831. doi: 10.1016/j.watres.2025.123831, 40412032

[64] Guzman-Otazo J Gonzales-Siles L Poma V Bengtsson-Palme J Thorell K Flach C-F . Diarrheal bacterial pathogens and multi-resistant Enterobacteria in the Choqueyapu River in La Paz, Bolivia. PLoS One. (2019) 14:e0210735. doi: 10.1371/journal.pone.0210735, 30640938 PMC6331111

[65] Hasani K Sadeghi H Vosoughi M Sardari M Manouchehrifar M Arzanlou M. Characterization of beta-lactamase producing Enterobacterales isolated from an urban community wastewater treatment plant in Iran. Iran J Microbiol. (2023) 15:521–32. doi: 10.18502/ijm.v15i4.13506, 38045715 PMC10692975

[66] Ho Y-N Tsai H-C Hsu B-M Chiou C-S. The Association of *Salmonella Enterica* from aquatic environmental and clinical samples in Taiwan. Sci Total Environ. (2018) 624:106–13. doi: 10.1016/j.scitotenv.2017.12.122, 29248701

[67] Hooban B Fitzhenry K O'Connor L Miliotis G Joyce A Chueiri A . A longitudinal survey of antibiotic-resistant Enterobacterales in the Irish environment, 2019-2020. Sci Total Environ. (2022) 828:154488. doi: 10.1016/j.scitotenv.2022.154488, 35278563

[68] Hooda Y Islam S Kabiraj R Rahman H Sarkar H da Silva KE . Old tools, new applications: use of environmental bacteriophages for typhoid surveillance and evaluating vaccine impact. PLoS Negl Trop Dis. (2024) 18:e0011822. doi: 10.1371/journal.pntd.0011822, 38358956 PMC10868810

[69] Hu L Xue JZ Wu HX. Composition and distribution of bacteria, pathogens, and antibiotic resistance genes at Shanghai port, China. WATER. (2024) 16:2569. doi: 10.3390/w16182569

[70] Huang X Toro M Reyes-Jara A Moreno-Switt AI Adell AD Oliveira CJB . Integrative genome-centric metagenomics for surface water surveillance: elucidating microbiomes, antimicrobial resistance, and their associations. Water Res. (2024) 264:122208. doi: 10.1016/j.watres.2024.122208, 39116611

[71] Jiménez-Belenguer A Santiago-Cuellar P Castillo MA Moreno Y Botella S Ferrús MA. "Study of dissemination and removal of multidrug resistant Salmonella in two sewage treatment plants from Comunitat Valenciana (Spain)" In: Mendez-Vilas E, editor. Microbes in applied research: Current ADVANCES and challenges. Singapore: World Scientific Publishing Co. Pte. Ltd. (2012). 2012.

[72] Jokinen CC Koot J Cole L Desruisseau A Edge TA Khan IUH . The distribution of *Salmonella enterica* serovars and subtypes insurface water from five agricultural regions across Canada. Water Res. (2015) 76:120–31. doi: 10.1016/j.watres.2015.02.038, 25799976

[73] Jokinen CC Schreier H Mauro W Taboada E Isaac-Renton JL Topp E . The occurrence and sources of *Campylobacter* spp., *Salmonella enterica* and *Escherichia coli* O157:H7 in the Salmon River, British Columbia, Canada. J Water Health. (2010) 8:374–86. doi: 10.2166/wh.2009.076, 20154400

[74] Kawabe H Manfio L Magana Pena S Zhou NA Bradley KM Chen C . Harnessing non-standard nucleic acids for highly sensitive Icosaplex (20-plex) detection of microbial threats for environmental surveillance. ACS Synth Biol. (2025) 14:470–84. doi: 10.1021/acssynbio.4c00619, 39898969 PMC11854376

[75] Khalefa HS Ahmed ZS Abdel-Kader F Ismail EM Elshafiee EA. Sequencing and phylogenetic analysis of the Stn gene of *Salmonella* species isolated from different environmental sources at Lake Qarun protectorate: the role of migratory birds and public health importance. Vet World. (2021) 14:2764–72. doi: 10.14202/vetworld.2021.2764-2772, 34903938 PMC8654765

[76] Khan HA Neyaz LA Malak HA Alshehri WA Elbanna K Organji SR . Diversity and antimicrobial susceptibility patterns of clinical and environmental *Salmonella enterica* serovars in Western Saudi Arabia. Folia Microbiol. (2024) 69:13. doi: 10.1007/s12223-024-01172-1, 38767834

[77] Kim NY Shi HJ Oh SS Gong YW Kwon MJ Eom JS . Wastewater knows pathogen spread: analysis of residential wastewater for infectious microorganisms including Sars-Cov-2. Infect Chemother. (2023) 55:214–25. doi: 10.3947/ic.2022.0152, 37038731 PMC10323530

[78] Klangnurak W Hinthong W Aue-umneoy D Yomla R. Assessment of bacterial community and other microorganism along the lam Takhong watercourse, Nakhon Ratchasima, Thailand. Curr Microbiol. (2025) 82:248. doi: 10.1007/s00284-025-04229-7, 40244481

[79] Kokkinos P Mandilara G Nikolaidou A Velegraki A Theodoratos P Kampa D . Performance of three small-scale wastewater treatment plants. A challenge for possible re use. Environ Sci Pollut Res. (2015) 22:17744–52. doi: 10.1007/s11356-015-4988-3, 26154042

[80] Kraft AL Wells JE Frye JG Ibekwe AM Durso LM Hiott L . A comparison of methods to detect low levels of *Salmonella enterica* in surface waters to support antimicrobial resistance surveillance efforts performed in multiple laboratories. Sci Total Environ. (2023) 905:167189. doi: 10.1016/j.scitotenv.2023.167189, 37748604

[81] Krzyzanowski F Zappelini L Martone-Rocha S Dropa M Matté MH Nacache F . Quantification and characterization of *Salmonella* spp. isolates in sewage sludge with potential usage in agriculture. BMC Microbiol. (2014) 14:263. doi: 10.1186/s12866-014-0263-x 25927729 PMC4207900

[82] Kuhn KG Shukla R Mannell M Graves GM Miller AC Vogel J . Using wastewater surveillance to monitor gastrointestinal pathogen infections in the state of Oklahoma. Microorganisms. (2023) 11:2193. doi: 10.3390/microorganisms11092193, 37764037 PMC10536226

[83] LeBoa C Shrestha S Shakya J Naga SR Shrestha S Shakya M . Environmental sampling for Typhoidal salmonellas in household and surface waters in Nepal identifies potential transmission pathways. PLoS Negl Trop Dis. (2023) 17:e0011341. doi: 10.1371/journal.pntd.0011341, 37851667 PMC10615262

[84] Li NN Li MH Chen P Wood A Hilton J Zhou QY . Mapping bacterial diversity and antibiotic resistance across wastewater treatment plant stages: insights from high-resolution 16s Rrna sequencing of the V3-V4 regions to detection of multi-drug resistant bacteria. J Water Process Eng. (2025) 71:107143. doi: 10.1016/j.jwpe.2025.107143

[85] Liu P Ibaraki M Kapoor R Amin N Das A Miah R . Development of Moore swab and ultrafiltration concentration and detection methods for *Salmonella* Typhi and *Salmonella Paratyphi* a in wastewater and application in Kolkata, India and Dhaka, Bangladesh. Front Microbiol. (2021) 12:684094. doi: 10.3389/fmicb.2021.684094, 34335510 PMC8320291

[86] M'Ikanatha NM Goldblum ZS Cesari N Nawrocki EM Fu YZ Kovac J . Outbreak-associated *Salmonella* Baildon found in wastewater demonstrates how sewage monitoring can supplement traditional disease surveillance. J Clin Microbiol. (2024) 62:e00825–24. doi: 10.1128/jcm.00825-24, 39297648 PMC11481576

[87] Mafu NC Pironcheva G Okoh AI. Genetic diversity and in vitro antibiotic susceptibility profile of *Salmonella* species isolated from domestic water and wastewater sources in the eastern Cape Province of South Africa. Afr J Biotechnol. (2009) 8:1263–9.

[88] Malayil L Ramachandran P Chattopadhyay S Allard SM Bui A Butron J . Variations in bacterial communities and antibiotic resistance genes across diverse recycled and surface water irrigation sources in the mid-Atlantic and Southwest United States: a conserve two-year field study. Environ Sci Technol. (2022) 56:15019–33. doi: 10.1021/acs.est.2c02281, 36194536 PMC9632240

[89] Masarikova M Manga I Cizek A Dolejska M Oravcova V Myskova P . *Salmonella Enterica* resistant to antimicrobials in wastewater effluents and black-headed gulls in the Czech Republic, 2012. Sci Total Environ. (2016) 542:102–7. doi: 10.1016/j.scitotenv.2015.10.069, 26519571

[90] Meena B Anburajan L Selvaganapathi K Vinithkumar NV Dharani G. Characteristics and dynamics of *Salmonella* diversity and prevalence of biomarker genes in Port Blair bays, south Andaman, India. Mar Pollut Bull. (2020) 160:111582. doi: 10.1016/j.marpolbul.2020.111582, 32882603

[91] Mendoza-Guido B Barrantes K Rodríguez C Rojas-Jimenez K Arias-Andres M. The impact of urban pollution on plasmid-mediated resistance acquisition in Enterobacteria from a tropical river. Antibiotics. (2024) 13:1089. doi: 10.3390/antibiotics13111089, 39596782 PMC11591392

[92] Mondal L Hossain T Saha ML. Bacterial load, multiple antibiotic-resistance patterns, and cytotoxic effects of coliform and coliform-related bacteria associated with the surface water of Dhaka City. Bangladesh J Bot. (2024) 53:41–8. doi: 10.3329/bjb.v53i1.72298

[93] Moriñigo MA Cornax R Castro D Jimenez-Notaro M Romero P Borrego JJ. Antibiotic resistance of Salmonella strains isolated from natural polluted waters. J Appl Bacteriol. (1990) 68:297–302. doi: 10.1111/j.1365-2672.1990.tb02578.x, 2341328

[94] Odjadjare EC Olaniran AO. Prevalence of antimicrobial resistant and virulent Salmonella Spp. in treated effluent and receiving aquatic milieu of wastewater treatment plants in Durban, South Africa. Int J Environ Res Public Health. (2015) 12:9692–713. doi: 10.3390/ijerph120809692, 26295245 PMC4555307

[95] Okorie CN Emencheta SC Ezeugwu DN Iganga JC Nkereuwem RN Ozioko CC . Molecular characterization and resistance profiling of multidrug-resistance *Salmonella* species isolated from southeast Nigerian River. Trop J Nat Prod Res. (2024) 8:7006–11. doi: 10.26538/tjnpr/v8i4.36

[96] Olawale SI Busayo O-OM Olatunji OI Mariam M Olayinka OS. Plasmid profiles and antibiotic susceptibility patterns of bacteria isolated from abattoirs wastewater within Ilorin, Kwara, Nigeria. Iran J Microbiol. (2020) 12:547–55. doi: 10.18502/ijm.v12i6.5029, 33613909 PMC7884279

[97] Onuoha SC. The prevalence of antibiotic resistant Diarrhogenic bacterial species in surface waters, south eastern Nigeria. Ethiop J Health Sci. (2017) 27:319–30. doi: 10.4314/ejhs.v27i4.3, 29217934 PMC5615021

[98] Ooms D de Vries A Koedijk FD Generaal E Friesema IH Rouvroye M . Large outbreak of typhoid fever on a river cruise ship used as accommodation for asylum seekers, the Netherlands, 2022. Euro Surveill. (2024) 29:2300211. doi: 10.2807/1560-7917.ES.2024.29.5.2300211, 38304948 PMC10835751

[99] Pignato S Coniglio MA Faro G Lefevre M Weill F-X Giammanco G. Molecular epidemiology of ampicillin resistance in Salmonella Spp. and *Escherichia Coli* from wastewater and clinical specimens. Foodborne Pathog Dis. (2010) 7:945–51. doi: 10.1089/fpd.2009.0504, 20367333

[100] Rahim K Nawaz MN Almehmadi M Alsuwat MA Liu L Yu CY . Public health implications of antibiotic resistance in sewage water: an epidemiological perspective. Bioresour Bioprocess. (2024) 11:91. doi: 10.1186/s40643-024-00807-y, 39340706 PMC11438758

[101] Rigby J Elmerhebi E Diness Y Mkwanda C Tonthola K Galloway H . Optimized methods for detecting *Salmonella Typhi* in the environment using validated field sampling, culture and confirmatory molecular approaches. J Appl Microbiol. (2022) 132:1503–17. Epub 20210824. doi: 10.1111/jam.15237, 34324765

[102] Salih H Karaynir A Yalcin M Oryasin E Holyavkin C Basbulbul G . Metagenomic analysis of wastewater Phageome from a University Hospital in Turkey. Arch Microbiol. (2022) 204:353. doi: 10.1007/s00203-022-02962-2, 35637399

[103] Santiago P Jiménez-Belenguer A García-Hernández J Estellés RM Hernández Pérez M Castillo López MA . High prevalence of Salmonella spp. in wastewater reused for irrigation assessed by molecular methods. Int J Hyg Environ Health. (2018) 221:95–101. doi: 10.1016/j.ijheh.2017.10.007, 29107574

[104] Sarekoski A Lipponen A Hokajärvi A-M Räisänen K Tiwari A Paspaliari D . Simultaneous biomass concentration and subsequent quantitation of multiple infectious disease agents and antimicrobial resistance genes from community wastewater. Environ Int. (2024) 191:108973. doi: 10.1016/j.envint.2024.108973, 39182255

[105] Schwartzbrod J Block JC Collomb J. Surface water salmonellae: serotypes and antibiotic resistance. Arch Roum Pathol Exp Microbiol. (1983) 42:179–89. 6673696

[106] Shinohara N Tanaka H Saito T Deguchi J Kondo R Soda K . Detection of carriers of typhoid Bacilli by sewerage-tracing surveillance in Matsuyama City. Jpn J Med Sci Biol. (1981) 34:385–92. doi: 10.7883/yoken1952.34.385, 7334709

[107] Shinohara N Tanaka H Saito T Deguchi J Soda K Sugiyama T . Surveillance for typhoid fever in Matsuyama City during 1974-1981 and detection of *Salmonella Typhi* in sewage and river waters. Jpn J Med Sci Biol. (1983) 36:191–7. doi: 10.7883/yoken1952.36.191, 6632352

[108] Shrestha P Prasai Joshi T Nhemhaphuki S Sitoula K Maharjan J Ranjit R . Occurrence of antibiotic-resistant bacteria and their genes in Bagmati River, Nepal. Water Air Soil Pollut. (2023) 234:475. doi: 10.1007/s11270-023-06499-y

[109] Shrestha S Da Silva KE Shakya J Yu AT Katuwal N Shrestha R . Detection of *Salmonella Typhi* bacteriophages in surface waters as a scalable approach to environmental surveillance. PLoS Negl Trop Dis. (2024) 18:e0011912. doi: 10.1371/journal.pntd.0011912, 38329937 PMC10852241

[110] Shrestha S Malla B Haramoto E. High-throughput microfluidic quantitative Pcr system for the simultaneous detection of antibiotic resistance genes and bacterial and viral pathogens in wastewater. Environ Res. (2024) 255:119156. doi: 10.1016/j.envres.2024.119156, 38759773

[111] Shrestha S Malla B Haramoto E. 6-plex crystal digital Pcr® for comprehensive surveillance of respiratory and foodborne bacterial pathogens in wastewater. Environ Pollut. (2025) 375:126298. doi: 10.1016/j.envpol.2025.126298, 40274213

[112] Siqueira JAM Teixeira DM Da Piedade GJL Souza CDO Moura TCF de Nazare Miranda Bahia M . Environmental health of water bodies from a Brazilian Amazon metropolis based on a conventional and metagenomic approach. J Appl Microbiol. (2024) 135:lxae101. doi: 10.1093/jambio/lxae101, 38627246

[113] Skariyachan S Lokesh P Rao R Kumar AU Vasist KS Narayanappa R. A pilot study on water pollution and characterization of multidrug-resistant superbugs from Byramangala tank, Ramanagara District, Karnataka, India. Environ Monit Assess. (2013) 185:5483–95. doi: 10.1007/s10661-012-2961-x, 23114918

[114] Song Q Zhang D Gao H Wu J. Salmonella species' persistence and their high level of antimicrobial resistance in flooded man-made Rivers in China. Microb Drug Resist. (2018) 24:1404–11. doi: 10.1089/mdr.2017.0316, 29750591

[115] Sthapit N Malla B Tandukar S Thakali O Sherchand JB Haramoto E. Evaluating acute gastroenteritis-causing pathogen reduction in wastewater and the applicability of river water for wastewater-based epidemiology in the Kathmandu Valley, Nepal. Sci Total Environ. (2024) 919:170764. doi: 10.1016/j.scitotenv.2024.170764, 38331291

[116] Suzuki Y Ushijima M. Distribution of antimicrobial resistant Salmonella in an urban river that flows through the Provincial City of Miyazaki, Japan. Water Environ J. (2016) 30:290–7. doi: 10.1111/wej.12194

[117] Tajammul A Benson S Ahmed J VanDerslice J Tanner WD. Detection of Salmonella Typhi and Blactx-M genes in drinking water, wastewater, and environmental biofilms in Sindh Province, Pakistan. PLoS Negl Trop Dis. (2025) 19:e0012963. doi: 10.1371/journal.pntd.0012963, 40261919 PMC12077766

[118] Tesfaye H Alemayehu H Desta AF Eguale T. Antimicrobial susceptibility profile of selected Enterobacteriaceae in wastewater samples from health facilities, abattoir, downstream rivers and a Wwtp in Addis Ababa, Ethiopia. Antimicrob Resist Infect Control. (2019) 8:134. doi: 10.1186/s13756-019-0588-1, 31413825 PMC6688205

[119] Toyting J Nuanmuang N Utrarachkij F Supha N Thongpanich Y Leekitcharoenphon P . Genomic analysis of Salmonella isolated from canal water in Bangkok, Thailand. Microbiol Spectr. (2024) 12:e04216–23. doi: 10.1128/spectrum.04216-23, 38563788 PMC11064549

[120] Uzzell CB Gray E Rigby J Troman CM Diness Y Mkwanda C . Environmental surveillance for *Salmonella Typhi* in rivers and wastewater from an informal sewage network in Blantyre, Malawi. PLoS Negl Trop Dis. (2024) 18:e0012518. doi: 10.1371/journal.pntd.0012518, 39331692 PMC11463779

[121] Viancelli A Deuner CW Rigo M Padilha J Marchesi JAP Fongaro G. Microbiological quality and genotoxic potential of surface water located above the Guarani aquifer. Environ Earth Sci. (2015) 74:5517–23. doi: 10.1007/s12665-015-4561-x

[122] Victoria NS Sree Devi Kumari T Lazarus B. Assessment on impact of sewage in coastal pollution and distribution of fecal pathogenic bacteria with reference to antibiotic resistance in the coastal area of cape Comorin, India. Mar Pollut Bull. (2022) 175:113123. doi: 10.1016/j.marpolbul.2021.113123, 34872749

[123] Victoria TNS Kumari TSD Lazarus B. Spatial distribution of faecal indicator bacteria around Kanyakumari coast, southernmost point of mainland India. Reg Stud Mar Sci. (2024) 77:13. doi: 10.1016/j.rsma.2024.103704

[124] Vincent V Scott HM Harvey RB Alali WQ Hume ME. Novel surveillance of *Salmonella Enterica* serotype Heidelberg epidemics in a closed community. Foodborne Pathog Dis. (2007) 4:375–85. doi: 10.1089/fpd.2007.0025, 17883321

[125] Xi X Zhang J Kwok L Huo D Feng S Zhang H . Microbial pollution tracking of dairy farm with a combined PCR-DGGE and qPCR approach. Curr Microbiol. (2015) 71:678–86. doi: 10.1007/s00284-015-0887-6, 26341923

[126] Zhang CM Xu LM Mou X Xu H Liu J Miao YH . Characterization and evolution of antibiotic resistance of *Salmonella* in municipal wastewater treatment plants. J Environ Manag. (2019) 251:8. doi: 10.1016/j.jenvman.2019.109547 31539702

[127] Rosofsky AS Vorhees DJ. Bringing multisectoral and multidisciplinary stakeholders together to optimize environmental Health Research. GeoHealth. (2023) 7:e2022GH000746. doi: 10.1029/2022GH000746, 36825115 PMC9941472

[128] Milazzo A Liu J Multani P Steele S Hoon E Chaber A-L. One health implementation: a systematic scoping review using the quadripartite one health joint plan of action. One Health. (2025) 20:101008. doi: 10.1016/j.onehlt.2025.101008, 40160937 PMC11953970

[129] Hyun C Burt Z Crider Y Nelson KL Prasad CSS Rayasam SDG . Sanitation for low-income regions: a cross-disciplinary review. Annu Rev Environ Resour. (2019) 44:287–318. doi: 10.1146/annurev-environ-101718-033327, 32587484 PMC7316187

[130] Sinharoy SS Pittluck R Clasen T. Review of drivers and barriers of water and sanitation policies for urban informal settlements in low-income and middle-income countries. Util Policy. (2019) 60:100957. doi: 10.1016/j.jup.2019.100957, 32214692 PMC7067261

[131] Furumai H. Rainwater and reclaimed wastewater for sustainable urban water use. Physics and Chemistry of the Earth, Parts A/B/C. (2008) 33:340–6. doi: 10.1016/j.pce.2008.02.029

[132] Tortajada C. Contributions of recycled wastewater to clean water and sanitation sustainable development goals. NPJ Clean Water. (2020) 3:22. doi: 10.1038/s41545-020-0069-3

[133] Ramm K Sielska M. The use of reclaimed water in the local urban cycle – a case study. Desalin Water Treat. (2023) 305:52–9. doi: 10.5004/dwt.2023.29525

[134] Qi Y Zhong Y Luo L He J Feng B Zhang X . Feasibility analysis of reclaimed water reuse based on water quality data and microbial community structure study. Sci Total Environ. (2024) 951:174781. doi: 10.1016/j.scitotenv.2024.174781, 39094655

[135] Heyde BJ Braun M Soufi L Lüneberg K Gallego S Amelung W . Transition from irrigation with untreated wastewater to treated wastewater and associated benefits and risks. NPJ Clean Water. (2025) 8:6. doi: 10.1038/s41545-025-00438-6

[136] Kumar A Goyal K. "Chapter two - water reuse in India: current perspective and future potential" In: Verlicchi P, editor. Advances in chemical pollution, environmental management and protection, vol. 6. London, UK: Elsevier (2020). 33–63.

[137] Qiu J Shen Z Leng G Wei G. Synergistic effect of drought and rainfall events of different patterns on watershed systems. Sci Rep. (2021) 11:18957. doi: 10.1038/s41598-021-97574-z, 34556685 PMC8460717

[138] Moore B. Typhoid: epidemiological investigation and control measures. Public Health. (1971) 85:152–8. doi: 10.1016/S0033-3506(71)80054-9, 5091060

[139] Andrews JR Yu AT Saha S Shakya J Aiemjoy K Horng L . Environmental surveillance as a tool for identifying high-risk settings for typhoid transmission. Clin Infect Dis. (2020) 71:S71–8. doi: 10.1093/cid/ciaa513, 32725227 PMC7446943

[140] Singh S Ahmed AI Almansoori S Alameri S Adlan A Odivilas G . A narrative review of wastewater surveillance: pathogens of concern, applications, detection methods, and challenges. Front Public Health. (2024) 12:1445961. doi: 10.3389/fpubh.2024.1445961, 39139672 PMC11319304

[141] Pang J Wong JCC Wulandari SM Tay M Karlsson EA Oktaria V . Wastewater surveillance for early pathogen detection in Asia. Int J Environ Health Res. (2025):1–10. doi: 10.1080/09603123.2025.2544736, 40828164

[142] Li J Hosegood I Powell D Tscharke B Lawler J Thomas KV . A global aircraft-based wastewater genomic surveillance network for early warning of future pandemics. Lancet Glob Health. (2023) 11:e791–5. doi: 10.1016/S2214-109X(23)00129-8, 37061316 PMC10101754

[143] Jones DL Bridgman M Pellett C Weightman AJ Kille P García Delgado Á . Use of wastewater from passenger ships to assess the movement of COVID-19 and other pathogenic viruses across maritime international boundaries. Front Public Health. (2024) 12:1377996. doi: 10.3389/fpubh.2024.1377996, 39076415 PMC11284076

[144] Morfino R Gawlik BM Tavazzi S Tessarolo A Gutierrez AB Madhav NK . Establishing a European wastewater pathogen monitoring network employing aviation samples: a proof of concept. Hum Genomics. (2025) 19:24. doi: 10.1186/s40246-025-00725-w, 40069832 PMC11900139

[145] St-Onge G Davis JT Hébert-Dufresne L Allard A Urbinati A Scarpino SV . Pandemic monitoring with global aircraft-based wastewater surveillance networks. Nat Med. (2025) 31:788–96. doi: 10.1038/s41591-025-03501-4, 39939526 PMC11922747

[146] Sikorski MJ Levine MM. Reviving the “Moore swab”: a classic environmental surveillance tool involving filtration of flowing surface water and sewage water to recover Typhoidal Salmonella Bacteria. Appl Environ Microbiol. (2020) 86:e00060–20. doi: 10.1128/AEM.00060-20, 32332133 PMC7301852

[147] Boulbes DR Costello T Baggerly K Fan F Wang R Bhattacharya R . A survey on data reproducibility and the effect of publication process on the ethical reporting of laboratory research. Clin Cancer Res. (2018) 24:3447–55. doi: 10.1158/1078-0432.Ccr-18-0227, 29643062 PMC6050098

[148] Westgard JO Westgard SA. Quality control review: implementing a scientifically based quality control system. Ann Clin Biochem. (2016) 53:32–50. doi: 10.1177/0004563215597248, 26150675

[149] Fowotade A Fayemiwo S Bongomin F Fasuyi T Aigbovo O Adegboro B. Internal and external quality control in the medical microbiology laboratory. Afr J Clin Exp Microbiol. (2018) 19:238–50. doi: 10.4314/ajcem.v19i4.1

[150] Badrick T. Integrating quality control and external quality assurance. Clin Biochem. (2021) 95:15–27. doi: 10.1016/j.clinbiochem.2021.05.003 33965412

[151] One Health High-Level Expert Panel Adisasmito WB Almuhairi S Behravesh CB Bilivogui P Bukachi SA . One health: a new definition for a sustainable and healthy future. PLoS Pathog. (2022) 18:e1010537. doi: 10.1371/journal.ppat.1010537, 35737670 PMC9223325

[152] World Health Organization. Typhoid vaccines: WHO position paper - march 2018. Wkly Epidemiol Rec. (2018) 93:153–72.

[153] World Health Organization. Typhoid vaccines: WHO position paper, march 2018 – recommendations. Vaccine. (2019) 37:214–6. doi: 10.1016/j.vaccine.2018.04.022, 29661581

[154] Kumar R Adeyemi NO Chattaraj S Alloun W Thamarsha AKANWMRK Anđelković S . Antimicrobial resistance in *Salmonella*: one health perspective on global food safety challenges. Science in One Health. (2025) 4:100117. doi: 10.1016/j.soh.2025.100117 40687400 PMC12274912

