# Supplementary material for: Systematic review on the laboratory methodology for conducting wastewater and environmental surveillance for Salmonella
Source: Front Public Health. 2026 Feb 23;14:1755256. doi: 10.3389/fpubh.2026.1755256 (PMC12968270; doi:10.3389/fpubh.2026.1755256)
Supplement: Supplementary file 3 [file Data_Sheet_1.docx]

Supplementary Material

**Contents**

[1 2020 PRISMA Checklist 2](#_Toc215077081)

[Supplementary Table 1 2](#_Toc215077082)

[Supplementary Table 2 5](#_Toc215077083)

[2 Search Strategy 6](#_Toc215077084)

[Supplementary Figure S1 6](#_Toc215077085)

[Supplementary Table 3 6](#_Toc215077086)

[Supplementary Table 4 7](#_Toc215077087)

[Supplementary Table 5 8](#_Toc215077088)

[Supplementary Figure 2 8](#_Toc215077089)

[Supplementary Table 6 9](#_Toc215077090)

[3 Inclusion and Exclusion Criteria 10](#_Toc215077091)

[Supplementary Table 7 10](#_Toc215077092)

[4 Structured Data Extraction Template 11](#_Toc215077093)

[4.1 Data Extraction Template 11](#_Toc215077094)

[Supplementary Table T8 11](#_Toc215077095)

[4.2 Quality Assessment Template 12](#_Toc215077096)

[Supplementary Table T9 12](#_Toc215077097)

[5 Supplementary Results 14](#_Toc215077098)

[Supplementary Table 10 14](#_Toc215077099)

[Supplementary Table 11 17](#_Toc215077100)

[Supplementary Figure 3 20](#_Toc215077101)

[Supplementary Figure 4 20](#_Toc215077102)

[Supplementary Figure 5 21](#_Toc215077103)

[Supplementary Figure 6 22](#_Toc215077104)

# 2020 PRISMA Checklist

Supplementary Table 1: PRISMA Checklist

| **Section and Topic** | **Item #** | **Checklist item** | **Location where item is reported** |
| --- | --- | --- | --- |
| **TITLE** | | | |
| Title | 1 | Identify the report as a systematic review. | Title |
| **ABSTRACT** | | | |
| Abstract | 2 | See the PRISMA 2020 for Abstracts checklist. | Supplementary Table T2 |
| **INTRODUCTION** | | | |
| Rationale | 3 | Describe the rationale for the review in the context of existing knowledge. | Introduction |
| Objectives | 4 | Provide an explicit statement of the objective(s) or question(s) the review addresses. | Introduction |
| **METHODS** | | | |
| Eligibility criteria | 5 | Specify the inclusion and exclusion criteria for the review and how studies were grouped for the syntheses. | Supplementary Table |
| Information sources | 6 | Specify all databases, registers, websites, organisations, reference lists and other sources searched or consulted to identify studies. Specify the date when each source was last searched or consulted. | Methods |
| Search strategy | 7 | Present the full search strategies for all databases, registers and websites, including any filters and limits used. | Methods, Supplementary file |
| Selection process | 8 | Specify the methods used to decide whether a study met the inclusion criteria of the review, including how many reviewers screened each record and each report retrieved, whether they worked independently, and if applicable, details of automation tools used in the process. | Methods |
| Data collection process | 9 | Specify the methods used to collect data from reports, including how many reviewers collected data from each report, whether they worked independently, any processes for obtaining or confirming data from study investigators, and if applicable, details of automation tools used in the process. | Methods |
| Data items | 10a | List and define all outcomes for which data were sought. Specify whether all results that were compatible with each outcome domain in each study were sought (e.g. for all measures, time points, analyses), and if not, the methods used to decide which results to collect. | Methods, Supplementary file |
|  | 10b | List and define all other variables for which data were sought (e.g. participant and intervention characteristics, funding sources). Describe any assumptions made about any missing or unclear information. | Supplementary Table T3 |
| Study risk of bias assessment | 11 | Specify the methods used to assess risk of bias in the included studies, including details of the tool(s) used, how many reviewers assessed each study and whether they worked independently, and if applicable, details of automation tools used in the process. | Methods |
| Effect measures | 12 | Specify for each outcome the effect measure(s) (e.g. risk ratio, mean difference) used in the synthesis or presentation of results. | Not applicable |
| Synthesis methods | 13a | Describe the processes used to decide which studies were eligible for each synthesis (e.g. tabulating the study intervention characteristics and comparing against the planned groups for each synthesis (item #5)). | Not applicable |
|  | 13b | Describe any methods required to prepare the data for presentation or synthesis, such as handling of missing summary statistics, or data conversions. | Not applicable |
|  | 13c | Describe any methods used to tabulate or visually display results of individual studies and syntheses. | Not applicable |
|  | 13d | Describe any methods used to synthesize results and provide a rationale for the choice(s). If meta-analysis was performed, describe the model(s), method(s) to identify the presence and extent of statistical heterogeneity, and software package(s) used. | Not applicable |
|  | 13e | Describe any methods used to explore possible causes of heterogeneity among study results (e.g. subgroup analysis, meta-regression). | Not applicable |
|  | 13f | Describe any sensitivity analyses conducted to assess robustness of the synthesized results. | Not applicable |
| Reporting bias assessment | 14 | Describe any methods used to assess risk of bias due to missing results in a synthesis (arising from reporting biases). | Not applicable |
| Certainty assessment | 15 | Describe any methods used to assess certainty (or confidence) in the body of evidence for an outcome. | Not applicable |
| **RESULTS** | | | |
| Study selection | 16a | Describe the results of the search and selection process, from the number of records identified in the search to the number of studies included in the review, ideally using a flow diagram. | Results |
|  | 16b | Cite studies that might appear to meet the inclusion criteria, but which were excluded, and explain why they were excluded. | Results |
| Study characteristics | 17 | Cite each included study and present its characteristics. | Results |
| Risk of bias in studies | 18 | Present assessments of risk of bias for each included study. | Supplementary file |
| Results of individual studies | 19 | For all outcomes, present, for each study: (a) summary statistics for each group (where appropriate) and (b) an effect estimate and its precision (e.g. confidence/credible interval), ideally using structured tables or plots. | Not applicable |
| Results of syntheses | 20a | For each synthesis, briefly summarise the characteristics and risk of bias among contributing studies. | Not applicable |
|  | 20b | Present results of all statistical syntheses conducted. If meta-analysis was done, present for each the summary estimate and its precision (e.g. confidence/credible interval) and measures of statistical heterogeneity. If comparing groups, describe the direction of the effect. | Not applicable |
|  | 20c | Present results of all investigations of possible causes of heterogeneity among study results. | Not applicable |
|  | 20d | Present results of all sensitivity analyses conducted to assess the robustness of the synthesized results. | Not applicable |
| Reporting biases | 21 | Present assessments of risk of bias due to missing results (arising from reporting biases) for each synthesis assessed. | Not applicable |
| Certainty of evidence | 22 | Present assessments of certainty (or confidence) in the body of evidence for each outcome assessed. | Not applicable |
| **DISCUSSION** | | | |
| Discussion | 23a | Provide a general interpretation of the results in the context of other evidence. | Discussion |
|  | 23b | Discuss any limitations of the evidence included in the review. | Discussion |
|  | 23c | Discuss any limitations of the review processes used. | Discussion |
|  | 23d | Discuss implications of the results for practice, policy, and future research. | Discussion |
| **OTHER INFORMATION** | | | |
| Registration and protocol | 24a | Provide registration information for the review, including register name and registration number, or state that the review was not registered. | Supplementary file |
|  | 24b | Indicate where the review protocol can be accessed, or state that a protocol was not prepared. | Supplementary file |
|  | 24c | Describe and explain any amendments to information provided at registration or in the protocol. | Not applicable |
| Support | 25 | Describe sources of financial or non-financial support for the review, and the role of the funders or sponsors in the review. | Other information/ Authors’ declarations |
| Competing interests | 26 | Declare any competing interests of review authors. | Other information/ Authors’ declarations |
| Availability of data, code and other materials | 27 | Report which of the following are publicly available and where they can be found: template data collection forms; data extracted from included studies; data used for all analyses; analytic code; any other materials used in the review. | Other information/ Authors’ declarations |

*From:*  Page MJ, McKenzie JE, Bossuyt PM, Boutron I, Hoffmann TC, Mulrow CD, et al. The PRISMA 2020 statement: an updated guideline for reporting systematic reviews. BMJ 2021;372:n71. doi: 10.1136/bmj.n71. This work is licensed under CC BY 4.0. To view a copy of this license, visit <https://creativecommons.org/licenses/by/4.0/>

Supplementary Table 2: PRISMA Checklist for Abstract

| **Section and Topic** | **Item #** | **Checklist item** | **Reported (Yes/No)** |
| --- | --- | --- | --- |
| **TITLE** | | |  |
| Title | 1 | Identify the report as a systematic review. | Yes |
| **BACKGROUND** | | |  |
| Objectives | 2 | Provide an explicit statement of the main objective(s) or question(s) the review addresses. | Yes |
| **METHODS** | | |  |
| Eligibility criteria | 3 | Specify the inclusion and exclusion criteria for the review. |  |
| Information sources | 4 | Specify the information sources (e.g. databases, registers) used to identify studies and the date when each was last searched. | Yes |
| Risk of bias | 5 | Specify the methods used to assess risk of bias in the included studies. |  |
| Synthesis of results | 6 | Specify the methods used to present and synthesise results. | Not applicable |
| **RESULTS** | | |  |
| Included studies | 7 | Give the total number of included studies and participants and summarise relevant characteristics of studies. | Yes |
| Synthesis of results | 8 | Present results for main outcomes, preferably indicating the number of included studies and participants for each. If meta-analysis was done, report the summary estimate and confidence/credible interval. If comparing groups, indicate the direction of the effect (i.e. which group is favoured). | Yes |
| **DISCUSSION** | | |  |
| Limitations of evidence | 9 | Provide a brief summary of the limitations of the evidence included in the review (e.g. study risk of bias, inconsistency and imprecision). |  |
| Interpretation | 10 | Provide a general interpretation of the results and important implications. | Yes |
| **OTHER** | | |  |
| Funding | 11 | Specify the primary source of funding for the review. |  |
| Registration | 12 | Provide the register name and registration number. |  |

*From:*  Page MJ, McKenzie JE, Bossuyt PM, Boutron I, Hoffmann TC, Mulrow CD, et al. The PRISMA 2020 statement: an updated guideline for reporting systematic reviews. BMJ 2021;372:n71. doi: 10.1136/bmj.n71. This work is licensed under CC BY 4.0. To view a copy of this license, visit <https://creativecommons.org/licenses/by/4.0/>

# Search Strategy


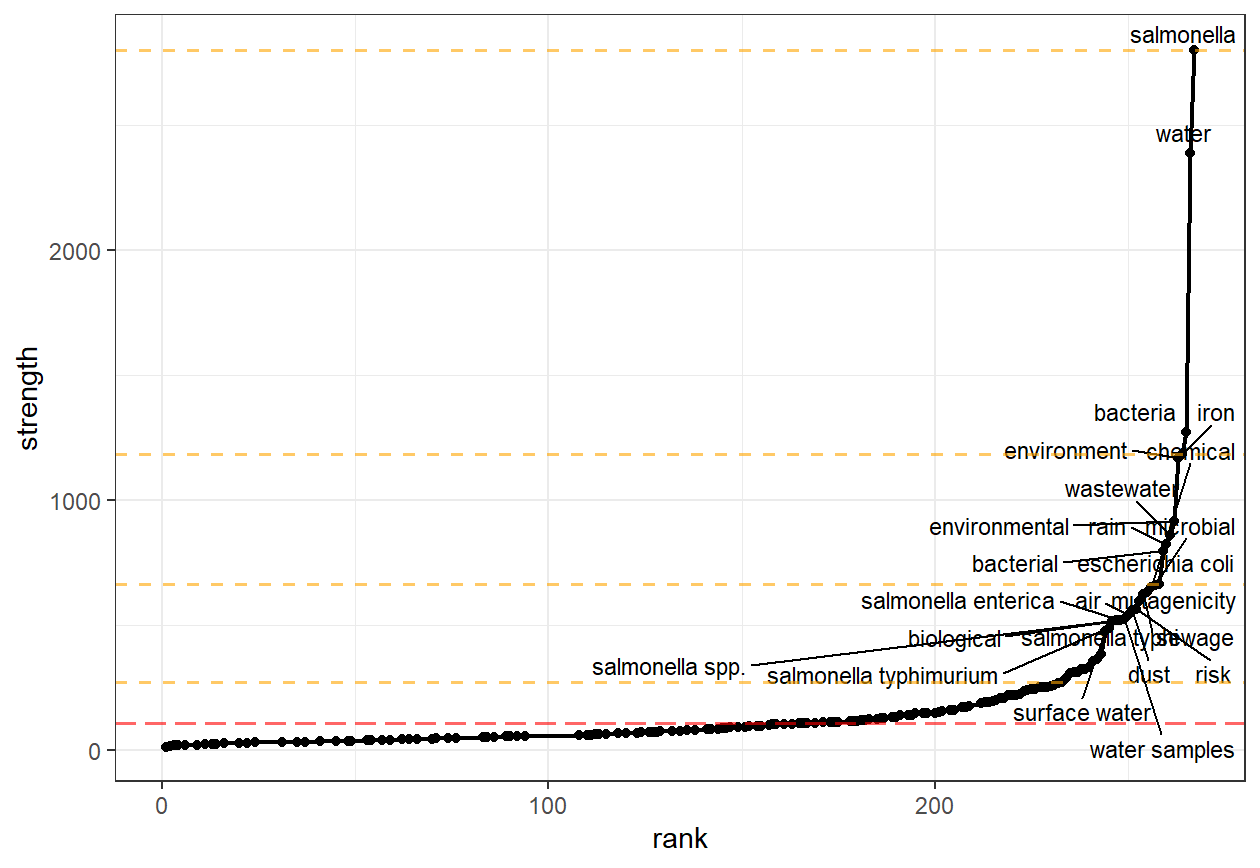


Supplementary Figure S1: Unbiased identification of keywords from naïve search in PubMed using easyPubMed and litsearchr packages in R.

Supplementary Table 3: Identified Keywords from naïve search

| salmonella enterica | salmonella typhi | salmonella typhimurium |
| --- | --- | --- |
| sewage sludge | surface water | treatment plant |
| wastewater treatment | water quality | water samples |
| agricultural | air | bacteria |
| bacterial | biological | chemical |
| Dust | e. coli | environment |
| environmental | escherichia coli | gel |
| genotoxicity | indicators | iron |
| microbial | mutagenicity | prevalence |
| Rain | risk | salmonella |
| salmonella spp. | Sewage | soil |
| wastewater | water |  |

Supplementary Table 4: Studies selected for optimization of PubMed search query

| **Title** | **Year** | **PMID** |
| --- | --- | --- |
| Detection of carriers of typhoid bacilli by sewerage-tracing surveillance in Matsuyama City | 1981 | 7334709 |
| Surveillance for typhoid fever in Matsuyama city during 1974-1981 and detection of Salmonella typhi in sewage and river waters | 1983 | 6632352 |
| Novel surveillance of Salmonella enterica serotype Heidelberg epidemics in a closed community | 2007 | 17883321 |
| Behaviour of pathogenic and indicator bacteria during urban wastewater treatment and sludge composting, as revealed by quantitative PCR | 2008 | 17659319 |
| Municipal Wastewater as a Microbial Surveillance Platform for Enteric Diseases: A Case Study for Salmonella and Salmonellosis | 2018 | 29630348 |
| Characterization and evolution of antibiotic resistance of Salmonella in municipal wastewater treatment plants | 2019 | 31539702 |
| Reviving the “Moore Swab”: a Classic Environmental Surveillance Tool Involving Filtration of Flowing Surface Water and Sewage Water To Recover Typhoidal <i>Salmonella</i> Bacteria | 2020 | 32332133 |
| Review of Methods Suitable for Environmental Surveillance of Salmonella Typhi and Paratyphi | 2020 | 32725228 |
| Characterization of Salmonella Isolates from Wastewater Treatment Plant Influents to Estimate Unreported Cases and Infection Sources of Salmonellosis | 2020 | 31936747 |
| Case-Control Study of Household and Environmental Transmission of Typhoid Fever in India | 2021 | 35238355 |
| Development of Moore Swab and Ultrafiltration Concentration and Detection Methods for Salmonella Typhi and Salmonella Paratyphi A in Wastewater and Application in Kolkata, India and Dhaka, Bangladesh | 2021 | 34335510 |
| Using Wastewater Surveillance to Monitor Gastrointestinal Pathogen Infections in the State of Oklahoma | 2023 | 37764037 |
| Environmental sampling for typhoidal Salmonellas in household and surface waters in Nepal identifies potential transmission pathways | 2023 | 37851667 |
| Vibrio cholerae and Salmonella Typhi culture-based wastewater or non-sewered sanitation surveillance in a resource-limited region | 2024 | 38177335 |
| Large outbreak of typhoid fever on a river cruise ship used as accommodation for asylum seekers, the Netherlands, 2022 | 2024 | 38304948 |
| Detection of Salmonella Typhi bacteriophages in surface waters as a scalable approach to environmental surveillance | 2024 | 38329937 |
| Environmental Surveillance for Salmonella Typhi and its Association With Typhoid Fever Incidence in India and Malawi | 2024 | 37775091 |

Supplementary Table 5: Search queries used for optimization of search results. (Date of retrieval – September 2024)

| **SN** | **Query** | **Results** |
| --- | --- | --- |
|  | ("Salmonella" OR "Salmonella typhi" OR "Typhoid") AND ("Survei*" OR "environmental survei*" OR "wastewater survei*") AND ("Sewage" OR "Waste*") | 66 |
|  | ("Salmonella" OR "Salmonella typhi" OR "Typhoid Fever" or “pathogen”) AND ("Survei*" OR "environmental survei*" OR "wastewater survei*") AND ("Sewage" or "wastewater" OR "surface water" OR "environmental samp*") | 471 |
|  | ("Salmonella" OR "Salmonella typhi" OR "Typhoid Fever") AND ("Survei*" OR "environmental survei*" OR "wastewater survei*" or "Antibiotic*") AND ("Sewage*" or "wastewat*" OR "surface water" OR "environmental samp*" OR "WWTP*" OR "river" or "stream" or "drain") | 496 |
|  | ("Salmonella" OR "Salmonella typhi" OR "Typhoid Fever") AND ("Survei*" OR "environmental survei*" OR "wastewater survei*" OR "antibiotic resistance") AND ("Sewage*" OR "wastewat*" OR "surface water" OR "environmental samp*" OR "WWTP*" OR "river" OR "stream" OR "drain") | 355 |

Supplementary Figure 2: Optimization of PubMed search query to achieve 100% sensitivity for selected studies in supplementary Table T5.

Supplementary Table 6: Search queries used for searching the selected databases

| **Database** | **Search String** |
| --- | --- |
| **PubMed** | ("Salmonella" OR "Salmonella typhi" OR "Typhoid Fever") AND ("Survei*" OR "environmental survei*" OR "wastewater survei*" OR "antibiotic resistance") AND ("Sewage*" OR "wastewat*" OR "surface water" OR "environmental samp*" OR “wastewater treatment” OR "WWTP*" OR "river" OR "stream" OR "drain") |
| **Embase** | ('salmonella' OR 'salmonella typhi' OR 'typhoid fever') AND ('survei*' OR 'environmental survei*' OR 'wastewater survei*' OR 'antibiotic resistance') AND ('sewage*' OR 'wastewat*' OR 'surface water' OR 'environmental samp*' OR 'wastewater treatment' OR 'wwtp*' OR 'river' OR 'stream' OR 'drain') |
| **Web of Science** | TS=("Salmonella" OR "Salmonella typhi" OR "Typhoid Fever") AND TS=("Survei*" OR "environmental survei*" OR "wastewater survei*" OR "antibiotic resistance") AND TS=("Sewage*" OR "wastewat*" OR "surface water" OR "environmental samp*" OR "wastewater treatment" OR "WWTP*" OR "river" OR "stream" OR "drain") |

# Inclusion and Exclusion Criteria

Supplementary Table 7: Inclusion and exclusion criteria for the systematic review

|  | **Inclusion Criteria** | **Exclusion Criteria** |
| --- | --- | --- |
| Sample details | - Samples collected from wastewater or wastewater-contaminated water bodies resulting from human excrement containing Salmonella typhi. - Studies explaining laboratory methods, as mentioned below | - Samples collected from - freshwater sites, environmental sites (such as land or air), - veterinary sites (including animal rearing and animal production facilities) - contaminated food. - Studies not explaining laboratory methods. |
| Methodology | - Methodology-based articles for environmental surveillance of S.typhi provide descriptions for the detection or isolation of Salmonella from wastewater or wastewater-contaminated water sources from a community. | - No method for isolation or characterization of S. typhi from the sample |
| Type of studies | - Peer-reviewed research articles in the English language | - Articles belonging to the following categories will be excluded: - Pre-prints - Reviews, - Systematic or meta-reviews, - Lectures, - Opinion articles, - Perspectives, - News articles, or - Protocol submissions. |

# Structured Data Extraction Template

## Data Extraction Template

Supplementary Table T8: Structured data extraction template used for extracting the data. The extracted data is available from the following link (DOI to be shared).

| **S.No.** | **Data element** | **Details to be extracted** |
| --- | --- | --- |
| 1. | Sampling Sites | Type of sampling site   - Drain, wastewater treatment plant, rivers, tanks, others (specify) |
| 1.1 | Name of Country | Name of the country reporting the method   - Classification into LIC, LMIC, UMIC, and HIC based on the World Bank classification |
| 1.2 | Specific details (sites) | Any details specified by the authors on sampling sites |
| 2. | Sample type | Type(s) of sample(s) collected.   - Grab, trap, composite, Moore, other (specify) |
| 2.2 | Sample collection | Details of sample collection |
| 2.3 | Number of sampling sites | Number of sampling sites used for the study |
| 2.4 | Specific details (collection) | Any details specified by the authors on the sample collection |
| 3. | Sample handling | Sample handling and transportation   - Cold chain, no information, other (specify) |
| 3.1 | Specific details (handling) | Any detail specified by the authors on sample handling |
| 4. | Sample processing | Sample processing before sample testing in the lab   - Centrifugation, Filteration, Enrichment, PEG precipitation, no information, other (specify) |
| 4.1 | Specific details (processing) | Any details specified for processing the sample |
| 5. | Sample testing | Sample testing methodology   - Culture; Most Probable Number; Characterization – phenotypic (biotyping, serotyping, antimicrobial susceptibility, others), genotyping (molecular, other), genomics (sequencing, whole genome sequencing, metagenomics) |
| 5.1 | Specific details (testing) | Any details specified for testing the samples |
| 6. | Limitations | Limitations mentioned by the authors |

## Quality Assessment Template

Supplementary Table T9: Quality Assessment Template with assessment categories, questions used, and criteria for classifying the study as 'Yes', 'Partial', or 'No' on the evaluation criteria. The extracted data is available from the following link (DOI to be shared)

| **Category** | **Question** | **Guidance on “Yes”** | **Guidance on “Partial”** | **Guidance on “No”** |
| --- | --- | --- | --- | --- |
| Site selection | Q1. The study provided details of sampling sites. | The study outlines the details of sampling sites, including parameters such as location, catchment area, and population. | The study describes a few of the characteristics. | No information is provided. |
|  | Q2. The study has well-defined criteria for selecting the sampling site. | The study has rationalized site selection through a central question or hypothesis | The study provided some rationale for selection. | No rationale is provided. |
| Sample collection | Q3. The study provides details of sample collection. | The study has clearly defined sample collection details such as type, volume, and frequency | The study covered some sample collection details | No information is provided. |
| Sample handling and transport | Q4. The study provides details of sample handling and transport. | The study provides information on sample handling after collection, storage conditions, transport conditions, and transit times | The study covered some of the sample handling and transport details | No information is provided. |
| Sample processing | Q5. The study provided details of sample processing before laboratory testing. | The study provided detailed steps for sample processing (if used), OR no processing steps were used. | The study provided steps without details. | No information is provided. |
| Sample testing | Q6. The study provided a detailed description of the testing methodology | The study provided a detailed outline of the methods used | The study provided some details but did not capture all aspects. | No information was provided. |
|  | Q7. The study provided details about performing the laboratory testing | The study provided detailed information on conditions, kits, and reagents used for the testing, including any inferences used for interpretation | The study provided some details but did not capture all aspects. | No information was provided. |
|  | Q8. The study provided information on quality control or quality assurance aspects of laboratory testing | The study provided QC steps for all laboratory methods used | The study provided QC steps for some of the methods used | No information was provided. |

| **Scoring Criteria for Quality Assessment**  Q1 – Yes = 0.5, Partial = 0.25, No = 0  Q2 – Yes = 0.5, Partial = 0.25, No = 0  Q3 – Yes = 1.0, Partial = 0.5, No = 0  Q4 – Yes = 1.0, Partial = 0.5, No = 0  Q5 – Yes = 2.0, Partial = 1.0, No = 0  Q6 – Yes = 2.0, Partial = 1.0, No = 0  Q7 – Yes = 1.0, Partial = 0.5, No = 0  Q8 – Yes = 2.0, Partial = 1.0, No = 0 | **Methodology quality assessment criteria**  Score >=9 – Study methodology: Excellent  Score 8-9 – Study methodology: Robust  Score 7-8 – Study methodology: Good  Score 6-7 – Study methodology: Fair  Score <6 – Study methodology: Low |
| --- | --- |

# Supplementary Results

Supplementary Table 10: Clustering of reported methods into pathways based on the reporting country, sample, and steps of protocol used. Some papers used multiple samples and methods. Six pathways (P1-P6) were identified. Studies excluded from the analysis have been assigned pathway as “NA.”

| **S.No.** | **Paper ID** | **Method ID** | **Pathway** |
| --- | --- | --- | --- |
| 1 | Paper_001 | Method_001 | P5 |
|  |  | Method_002 | P1 |
| 2 | Paper_002 | Method_003 | P1 |
| 3 | Paper_003 | Method_004 | P2 |
| 4 | Paper_004 | Method_005 | P6 |
| 5 | Paper_005 | Method_006 | P2 |
| 6 | Paper_006 | Method_007 | P1 |
| 7 | Paper_007 | Method_008 | P3 |
| 8 | Paper_008 | Method_009 | P3 |
| 9 | Paper_009 | Method_010 | P2 |
| 10 | Paper_010 | Method_011 | NA |
| 11 | Paper_011 | Method_012 | NA |
| 12 | Paper_012 | Method_013 | P6 |
| 13 | Paper_013 | Method_014 | P2 |
| 14 | Paper_014 | Method_015 | P2 |
| 15 | Paper_015 | Method_016 | P2 |
| 16 | Paper_016 | Method_017 | P2 |
| 17 | Paper_017 | Method_018 | P2 |
| 18 | Paper_018 | Method_019 | P2 |
| 19 | Paper_019 | Method_020 | P2 |
| 20 | Paper_020 | Method_021 | P2 |
| 21 | Paper_021 | Method_022 | NA |
| 22 | Paper_022 | Method_023 | P2 |
| 23 | Paper_023 | Method_024 | P4 |
| 24 | Paper_024 | Method_025 | P4 |
| 25 | Paper_025 | Method_026 | P6 |
| 26 | Paper_026 | Method_027 | NA |
| 27 | Paper_027 | Method_028 | P2 |
| 28 | Paper_028 | Method_029 | P2 |
| 29 | Paper_029 | Method_030 | P2 |
| 30 | Paper_030 | Method_031 | NA |
| 31 | Paper_031 | Method_032 | NA |
| 32 | Paper_032 | Method_033 | P6 |
| 33 | Paper_033 | Method_034 | P5 |
| 34 | Paper_034 | Method_035 | P2 |
| 35 | Paper_035 | Method_036 | P2 |
| 36 | Paper_036 | Method_037 | P2 |
| 37 | Paper_037 | Method_038 | NA |
| 38 | Paper_038 | Method_039 | P2 |
| 39 | Paper_039 | Method_040 | P2 |
| 40 | Paper_040 | Method_041 | P5 |
| 41 | Paper_041 | Method_042 | P6 |
| 42 | Paper_042 | Method_043 | NA |
| 43 | Paper_043 | Method_044 | P2 |
| 44 | Paper_044 | Method_045 | NA |
| 45 | Paper_045 | Method_046 | P4 |
| 46 | Paper_046 | Method_047 | P5 |
| 47 | Paper_047 | Method_048 | P2 |
| 48 | Paper_048 | Method_049 | P1 |
|  |  | Method_050 | P5 |
| 49 | Paper_049 | Method_051 | P2 |
| 50 | Paper_050 | Method_052 | P6 |
| 51 | Paper_051 | Method_053 | P3 |
| 52 | Paper_052 | Method_054 | P2 |
| 53 | Paper_053 | Method_055 | P2 |
| 54 | Paper_054 | Method_056 | P4 |
| 55 | Paper_055 | Method_057 | P2 |
| 56 | Paper_056 | Method_058 | P2 |
| 57 | Paper_057 | Method_059 | P2 |
| 58 | Paper_058 | Method_060 | P4 |
| 59 | Paper_059 | Method_061 | P5 |
|  |  | Method_062 | P1 |
| 60 | Paper_060 | Method_063 | P2 |
| 61 | Paper_061 | Method_064 | P2 |
| 62 | Paper_062 | Method_065 | P2 |
| 63 | Paper_063 | Method_066 | P5 |
|  |  | Method_067 | P1 |
| 64 | Paper_064 | Method_068 | NA |
| 65 | Paper_065 | Method_069 | P2 |
| 66 | Paper_066 | Method_070 | P2 |
|  |  | Method_071 | P3 |
| 67 | Paper_067 | Method_072 | P6 |
| 68 | Paper_068 | Method_073 | P5 |
|  |  | Method_074 | P2 |
| 69 | Paper_069 | Method_075 | P4 |
| 70 | Paper_070 | Method_076 | P3 |
| 71 | Paper_071 | Method_077 | P3 |
| 72 | Paper_072 | Method_078 | P3 |
| 73 | Paper_073 | Method_079 | NA |
| 74 | Paper_074 | Method_080 | P5 |
| 75 | Paper_075 | Method_081 | NA |
| 76 | Paper_076 | Method_082 | P5 |
| 77 | Paper_077 | Method_083 | P5 |
| 78 | Paper_078 | Method_084 | P2 |
| 79 | Paper_079 | Method_085 | P2 |
| 80 | Paper_080 | Method_086 | P5 |
| 81 | Paper_081 | Method_087 | P2 |
| 82 | Paper_082 | Method_088 | P5 |
| 83 | Paper_083 | Method_089 | P2 |
| 84 | Paper_084 | Method_090 | NA |
| 85 | Paper_085 | Method_091 | P5 |
|  |  | Method_092 | P1 |
| 86 | Paper_086 | Method_093 | P5 |
|  |  | Method_094 | P1 |
| 87 | Paper_087 | Method_095 | NA |
| 88 | Paper_088 | Method_096 | P2 |
| 89 | Paper_089 | Method_097 | P2 |
| 90 | Paper_090 | Method_098 | P2 |
| 91 | Paper_091 | Method_099 | NA |
| 92 | Paper_092 | Method_100 | P4 |
| 93 | Paper_093 | Method_101 | P2 |
| 94 | Paper_094 | Method_102 | P2 |

Supplementary Table 11: Identification of domains from the title, abstracts, and keywords of manuscripts selected for extraction. Eight domains were identified: A) outbreak detection and investigation, B) disease prevalence, C) antimicrobial resistance prevalence, D) mechanisms of antimicrobial resistance, E) wastewater monitoring, F) environmental health, G) one health, and H) method validation.

| **Paper ID** | **A** | **B** | **C** | **D** | **E** | **F** | **G** | **H** |
| --- | --- | --- | --- | --- | --- | --- | --- | --- |
| Paper_001 | No | Yes | No | No | No | No | No | No |
| Paper_002 | No | Yes | No | No | Yes | Yes | No | No |
| Paper_003 | No | Yes | No | Yes | No | No | No | No |
| Paper_004 | No | No | No | No | No | Yes | No | No |
| Paper_005 | No | No | Yes | Yes | No | No | No | No |
| Paper_006 | No | No | No | No | No | Yes | Yes | No |
| Paper_007 | No | No | Yes | Yes | No | No | No | No |
| Paper_008 | No | Yes | No | No | No | Yes | No | No |
| Paper_009 | No | No | Yes | No | Yes | No | No | No |
| Paper_010 | No | Yes | No | No | Yes | Yes | No | No |
| Paper_011 | No | Yes | No | No | No | No | Yes | No |
| Paper_012 | No | No | No | No | No | Yes | No | No |
| Paper_013 | No | No | No | No | No | No | No | Yes |
| Paper_014 | No | No | Yes | No | No | Yes | No | No |
| Paper_015 | No | No | Yes | No | No | Yes | No | No |
| Paper_016 | Yes | Yes | No | No | No | No | No | No |
| Paper_017 | No | No | No | No | No | Yes | No | No |
| Paper_018 | No | No | Yes | Yes | No | No | No | No |
| Paper_019 | No | Yes | No | No | No | Yes | No | No |
| Paper_020 | No | Yes | Yes | No | No | No | Yes | No |
| Paper_021 | No | Yes | Yes | Yes | No | No | No | No |
| Paper_022 | No | No | Yes | No | No | Yes | No | No |
| Paper_023 | Yes | No | No | No | No | No | Yes | No |
| Paper_024 | Yes | No | No | No | No | No | No | No |
| Paper_025 | No | No | No | Yes | No | No | No | No |
| Paper_026 | No | Yes | Yes | No | No | Yes | No | No |
| Paper_027 | No | No | Yes | Yes | No | No | No | No |
| Paper_028 | No | Yes | No | No | No | No | No | No |
| Paper_029 | No | Yes | Yes | No | No | Yes | No | No |
| Paper_030 | Yes | Yes | No | No | No | No | No | No |
| Paper_031 | No | Yes | No | Yes | No | No | No | No |
| Paper_032 | No | Yes | No | No | No | Yes | No | No |
| Paper_033 | Yes | No | No | No | No | Yes | No | No |
| Paper_034 | No | No | No | No | Yes | No | No | No |
| Paper_035 | Yes | Yes | No | No | No | No | No | No |
| Paper_036 | No | Yes | Yes | No | No | No | Yes | No |
| Paper_037 | No | No | No | No | No | No | No | Yes |
| Paper_038 | No | No | No | No | No | No | Yes | No |
| Paper_039 | No | Yes | Yes | No | No | No | No | No |
| Paper_040 | Yes | No | No | No | No | No | Yes | Yes |
| Paper_041 | No | No | No | No | No | Yes | No | No |
| Paper_042 | No | No | No | No | Yes | No | No | No |
| Paper_043 | No | No | No | No | No | No | Yes | Yes |
| Paper_044 | No | Yes | Yes | No | Yes | No | Yes | No |
| Paper_045 | Yes | No | No | No | No | No | No | No |
| Paper_046 | No | No | No | No | No | Yes | No | No |
| Paper_047 | No | No | No | No | Yes | No | No | No |
| Paper_048 | No | No | No | No | No | No | No | Yes |
| Paper_049 | No | Yes | Yes | No | No | No | No | No |
| Paper_050 | No | No | No | No | Yes | Yes | No | No |
| Paper_051 | No | No | No | No | No | No | Yes | No |
| Paper_052 | No | No | No | No | No | Yes | No | No |
| Paper_053 | No | No | No | Yes | No | Yes | No | No |
| Paper_054 | Yes | No | No | No | No | No | No | No |
| Paper_055 | No | No | Yes | No | No | No | No | No |
| Paper_056 | No | No | Yes | No | No | No | No | No |
| Paper_057 | No | Yes | Yes | No | No | Yes | No | No |
| Paper_058 | No | Yes | Yes | No | No | Yes | No | No |
| Paper_059 | No | Yes | No | No | No | No | No | No |
| Paper_060 | No | No | Yes | Yes | No | Yes | No | No |
| Paper_061 | No | No | Yes | No | No | Yes | No | No |
| Paper_062 | Yes | No | No | No | No | No | No | No |
| Paper_063 | Yes | Yes | No | No | No | No | No | Yes |
| Paper_064 | No | Yes | Yes | Yes | No | No | No | No |
| Paper_065 | No | Yes | Yes | Yes | No | No | No | No |
| Paper_066 | No | No | No | No | No | No | No | Yes |
| Paper_067 | No | No | No | Yes | No | No | No | Yes |
| Paper_068 | No | No | No | No | Yes | No | No | Yes |
| Paper_069 | No | Yes | No | No | No | No | No | Yes |
| Paper_070 | No | No | Yes | No | No | No | No | No |
| Paper_071 | Yes | No | No | No | No | No | No | No |
| Paper_072 | Yes | No | No | No | No | Yes | No | No |
| Paper_073 | No | No | Yes | No | No | Yes | No | No |
| Paper_074 | No | No | No | No | No | No | No | Yes |
| Paper_075 | Yes | No | No | No | No | No | No | No |
| Paper_076 | No | No | No | No | No | No | No | Yes |
| Paper_077 | No | No | No | No | No | Yes | No | No |
| Paper_078 | No | No | No | No | No | Yes | No | No |
| Paper_079 | No | No | Yes | No | No | Yes | No | No |
| Paper_080 | Yes | No | No | No | No | No | No | No |
| Paper_081 | No | Yes | Yes | No | No | No | No | No |
| Paper_082 | Yes | No | No | No | No | No | No | No |
| Paper_083 | No | Yes | Yes | No | No | No | No | No |
| Paper_084 | Yes | No | No | Yes | No | No | No | No |
| Paper_085 | Yes | Yes | No | No | No | No | No | No |
| Paper_086 | No | Yes | No | No | No | No | No | No |
| Paper_087 | No | No | No | No | No | Yes | No | No |
| Paper_088 | No | Yes | Yes | No | No | No | No | No |
| Paper_089 | No | Yes | Yes | No | No | Yes | No | No |
| Paper_090 | Yes | No | No | No | No | No | No | No |
| Paper_091 | No | No | No | No | No | Yes | No | No |
| Paper_092 | Yes | No | No | No | No | No | No | No |
| Paper_093 | Yes | No | No | No | No | No | No | No |
| Paper_094 | No | No | Yes | No | No | No | No | No |


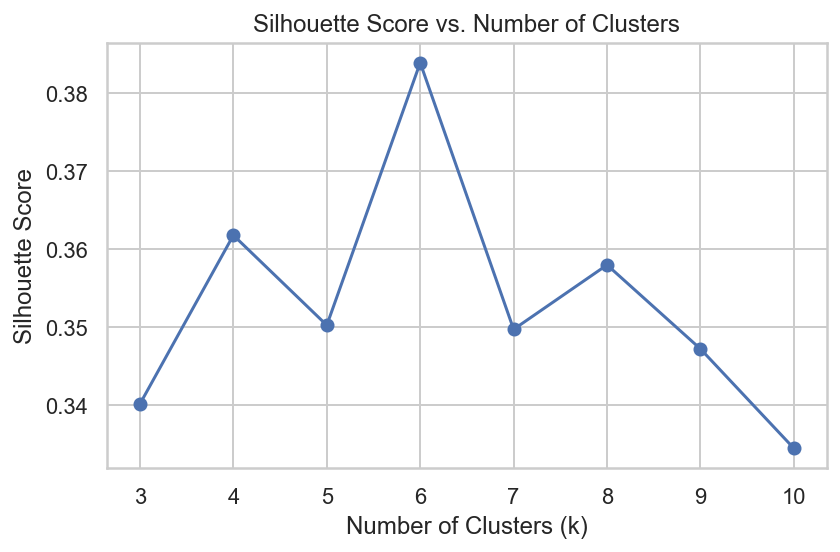

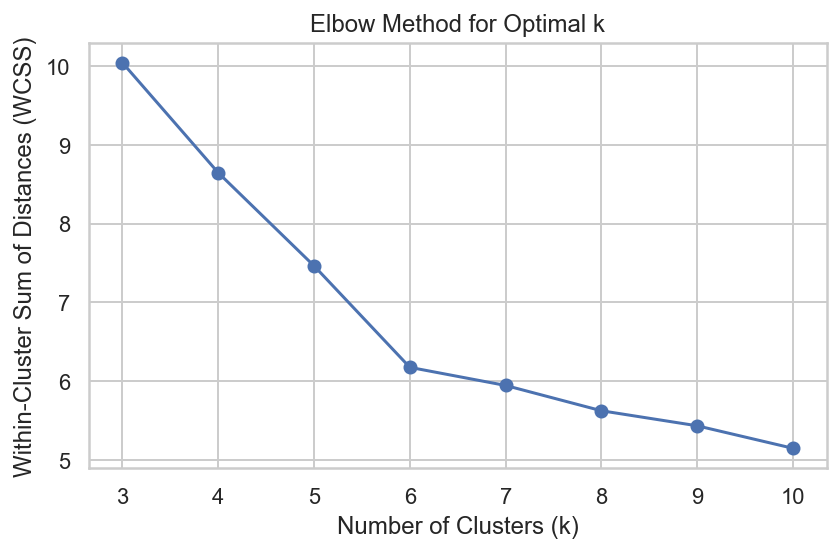


Supplementary Figure 3: A) Silhouette score; and B) Elbow method for determining the optimum number of clusters in the classification of methods to similar pathways.


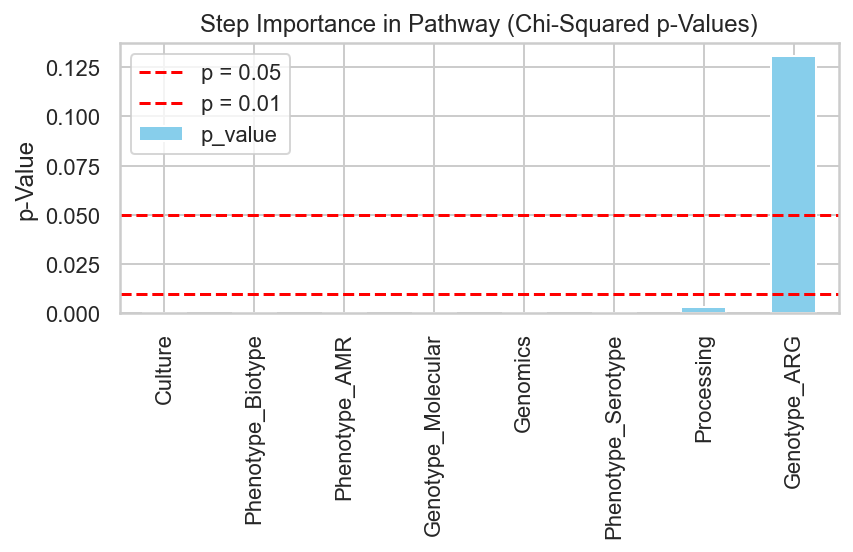


Supplementary Figure 4: Chi-squared p-values (at significance level of p=0.05 and 0.01) calculated by chi-squared test comparing the observed count of the presence and absence of steps in a pathway with expected counts under a null hypothesis of no difference.


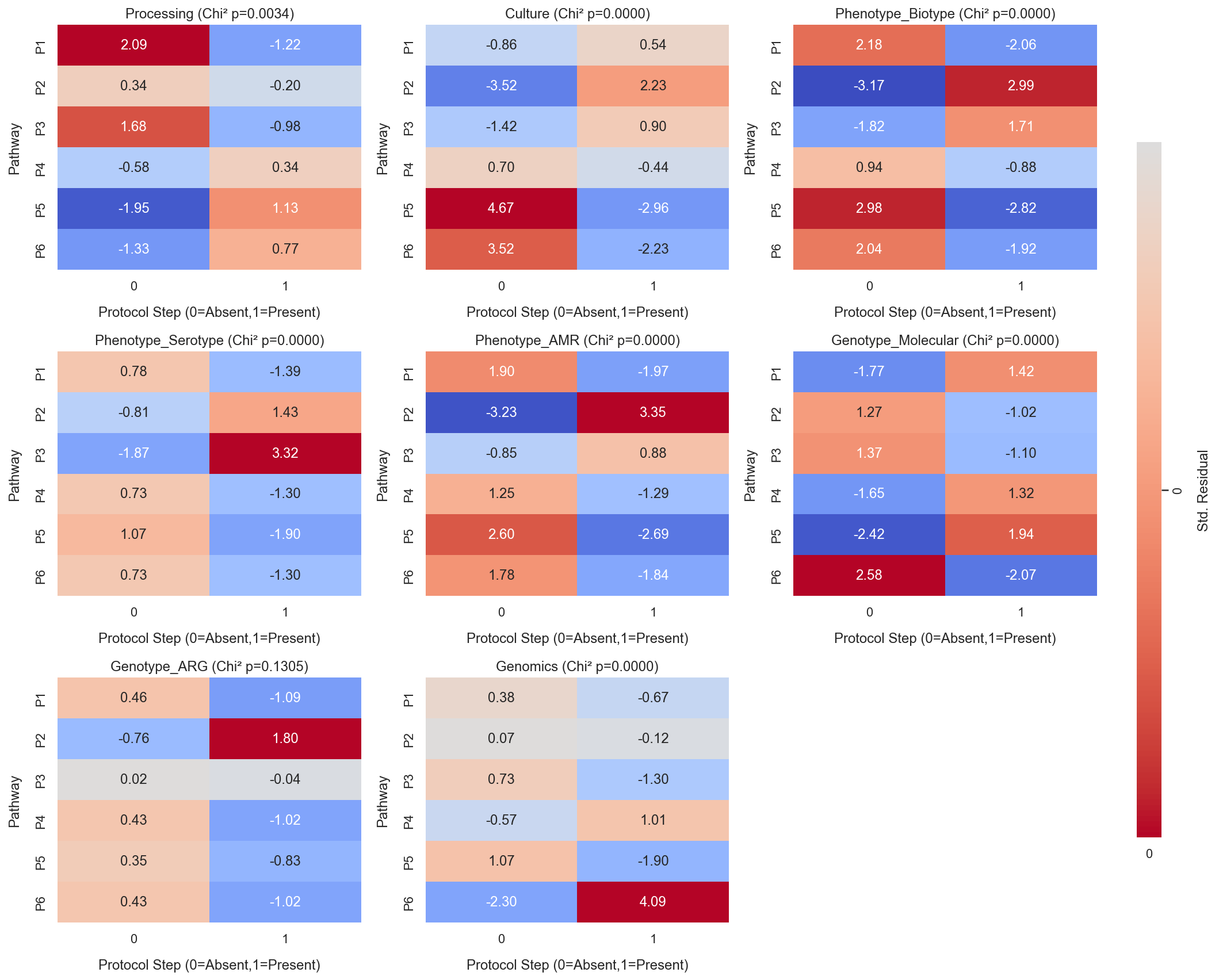


Supplementary Figure 5: post-hoc residual analysis of the chi-squared test across pathways. Residuals ≥ 2 indicate strong overrepresentation, meaning the pathway has more methods with the protocol step than expected under a random distribution. Conversely, residuals ≤ -2 indicate strong underrepresentation, meaning the pathway has fewer or no methods with the protocol step. A strong association of a step with one or more pathways can explain statistically significant p-values observed in the chi-squared test.

Supplementary Figure 6.The quality of studies was evaluated based on eight criteria. Q1. The study provides details of sampling sites (location, catchment area, population, etc.) [score: 0.5 for yes, 0.25 for partial], Q2. The study has well-defined criteria for selecting the sampling site (e.g., hypothesis or central question) [0.5 for yes, 0.25 for partial], Q3. The study provides details of sample collection (sample type, volume, and frequency) [1 for yes, 0.5 for partial], Q4. The study provides details of sample transport, including transport conditions and transit time [1 for yes, 0.5 for partial], Q5. The study provides details of sample pre-processing or processing before laboratory testing [2 for yes, 1 for partial], Q6. The study offers a detailed description of the testing methodology [2 for yes, 1 for partial], Q7. The study supplies details about conducting the laboratory testing [1 for yes, 0.5 for partial], Q8. The study includes information on quality control or quality assurance aspects of the laboratory testing method [2
